# Effects of extracorporeal CO_2_ removal on gas exchange and ventilator settings: a systematic review and meta-analysis

**DOI:** 10.1186/s13054-024-04927-x

**Published:** 2024-04-30

**Authors:** Alexandra-Maria Stommel, Harald Herkner, Calvin Lukas Kienbacher, Brigitte Wildner, Alexander Hermann, Thomas Staudinger

**Affiliations:** 1 Department of Emergency Medicine, Medical University of Vienna, Waehringer Guertel 18-20, 1090 Vienna, Austria; 2 University Library, Medical University of Vienna, Waehringer Guertel 18-20, 1090 Vienna, Austria; 3 Department of Medicine I, Intensive Care Unit 13i2, Medical University of Vienna, Waehringer Guertel 18-20, 1090 Vienna, Austria

**Keywords:** Acute respiratory distress syndrome (ARDS), Hypercapnic acidosis, Interventional lung assist, Extraction capacity

## Abstract

**Purpose:**

A systematic review and meta-analysis to evaluate the impact of extracorporeal carbon dioxide removal (ECCO_2_R) on gas exchange and respiratory settings in critically ill adults with respiratory failure.

**Methods:**

We conducted a comprehensive database search, including observational studies and randomized controlled trials (RCTs) from January 2000 to March 2022, targeting adult ICU patients undergoing ECCO_2_R. Primary outcomes were changes in gas exchange and ventilator settings 24 h after ECCO_2_R initiation, estimated as mean of differences, or proportions for adverse events (AEs); with subgroup analyses for disease indication and technology. Across RCTs, we assessed mortality, length of stay, ventilation days, and AEs as mean differences or odds ratios.

**Results:**

A total of 49 studies encompassing 1672 patients were included. ECCO_2_R was associated with a significant decrease in PaCO_2_, plateau pressure, and tidal volume and an increase in pH across all patient groups, at an overall 19% adverse event rate. In ARDS and lung transplant patients, the PaO_2_/FiO_2_ ratio increased significantly while ventilator settings were variable. “Higher extraction” systems reduced PaCO_2_ and respiratory rate more efficiently. The three available RCTs did not demonstrate an effect on mortality, but a significantly longer ICU and hospital stay associated with ECCO_2_R.

**Conclusions:**

ECCO_2_R effectively reduces PaCO_2_ and acidosis allowing for less invasive ventilation. “Higher extraction” systems may be more efficient to achieve this goal. However, as RCTs have not shown a mortality benefit but increase AEs, ECCO_2_R’s effects on clinical outcome remain unclear. Future studies should target patient groups that may benefit from ECCO_2_R.

*PROSPERO Registration No*: CRD 42020154110 (on January 24, 2021).

**Graphical abstract:**

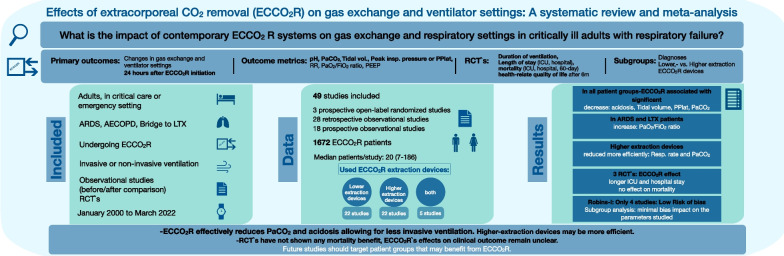

**Supplementary Information:**

The online version contains supplementary material available at 10.1186/s13054-024-04927-x.

## Introduction

Extracorporeal carbon dioxide removal (ECCO_2_R) implies the removal of carbon dioxide (CO_2_) from the blood across a gas exchange membrane without influencing oxygenation to a clinically relevant extent. ECCO_2_R can be provided by heterogenous techniques, thus the method has to be rather regarded as a therapeutic intention than as a specific technical procedure [1]. The term ECCO_2_R has been proposed first during the late seventies by Kolobow and Gattinoni in a study using an arterio-venous pumpless circuit in an animal model [2]. In 1986, Gattinoni`s group reported on patients suffering from severe acute respiratory distress syndrome (ARDS) undergoing ECCO_2_R by low-flow venovenous (VV) extracorporeal gas exchange to enable more protective ventilator settings [3]. The revival of ECCO_2_R was spurred by awareness of mechanical ventilation risks, aiming for ultraprotective ventilation with tidal volumes well below 6 mL/kg/predicted body weight. This led to the development of devices for CO_2_ removal, like the arterio-venous Interventional Lung Assist (ILA®, Novalung, Heilbronn, Germany), increasing its use in ARDS patients [4]. Reducing ventilation invasiveness in ARDS patients by ECCO_2_R has been the main therapeutic target under investigation to date [5, 6]. ECCO_2_R is used in hypercapnic lung failure, such as in acute exacerbated chronic obstructive pulmonary disease (AECOPD), targeting intubation avoidance or weaning, and in terminal fibrosis for lung transplantation (LTX) bridging, promoting spontaneous breathing and ambulation [1, 7–9]. As effective extracorporeal elimination of CO_2_ can be achieved at much lower blood flow than necessary for oxygenation, specially designed low-flow set-ups have been developed for the purpose of ECCO_2_R using smaller cannulas and membranes based either on continuous renal replacement therapy (CRRT) or on extracorporeal membrane oxygenation (ECMO) technology [10, 11].

The efficacy of CO_2_ removal depends on the partial pressure gradient at the membrane, the diffusion coefficient, the cross-sectional area of membrane lung, as well as blood flow and sweep gas flow [10, 12–14]. CO_2_ extraction tends to be less efficient in CRRT-based systems due to their lower blood flow and membrane surface, unlike ECMO-based systems with centrifugal pumps and larger membranes. A post hoc analysis of the SUPERNOVA trial [6] investigating the effects of ECCO_2_R in patients with moderate ARDS compared the subgroups treated with “lower extraction” and “higher extraction” systems [15]. Although the goal of reduced tidal volumes could be achieved in both groups, this was more frequently the case in the “higher extraction” group.

The therapeutic goal of ECCO_2_R depends on indication: In ARDS, it is to enable less invasive ventilation; in chronic obstructive pulmonary disease (COPD) or LTX bridging, it aims to reduce ventilatory strain, promoting spontaneous breathing or avoiding mechanical ventilation.

No systematic analysis of ECCO_2_R`s clinical effects, varying by indication and technology, exists yet. We conducted a systematic review to assess its effects on gas exchange and respiratory settings dependent on the different indications and its extraction capacity (“higher” versus “lower”).

## Methods

The review protocol was registered on PROSPERO (Registration No: CRD 42020154110) on 24th January 2021. The reporting adhered to the PRISMA guidelines [16]. The PRISMA checklist is provided as Additional file 1: File A.

Our objective was to examine the effect of contemporary ECCO_2_R systems on gas exchange and respiratory settings in critically ill adults, and in subgroups defined by technology and indications.

### Criteria for considering studies for this review

#### Types of studies

We included observational studies with at least a before-after comparison and randomized controlled trials. Only studies published after the year 2000 were considered to focus on contemporary ECCO_2_R systems. Animal studies were not included. We excluded abstracts, editorials, case reports, and case series with fewer than 10 subjects, and reviews. We did not impose any language restrictions.

#### Participant criteria

We focused on adult patients (≥ 18 years of age) in critical or emergency care settings undergoing ECCO_2_R who had respiratory failure conditions such as ARDS, AECOPD, or were bridged to LTX. These patients could be either on invasive or non-invasive ventilation (NIV).

#### Intervention types

Our primary goal was to evaluate the effects of contemporary extracorporeal CO_2_ removal systems on CO_2_ blood levels. Systems primarily designed for oxygenation (ECMO) were not included.

The criteria for considering studies to this review is available in the Additional file 2: File B.

#### Outcome measures

We focused on six specific outcome metrics with respect to ventilation (peak inspiratory or plateau pressure, positive end-expiratory pressure (PEEP), tidal volume, respiratory rate, arterial blood CO_2_ concentration (PaCO_2_), arterial blood CO_2_ to fraction of inspired oxygen ratio (PaO_2_/FiO_2_ ratio), and pH). Primary outcomes were changes in gas exchange and ventilatory settings within the first 24 h of ECCO_2_R initiation. If a study presented results at a different time frame, we selected the data point closest to the 24-h mark. Peak inspiratory or plateau pressure, tidal volume, PaCO_2_, and pH were regarded as important outcomes, while respiratory rate, PEEP, and PaO_2_/FiO_2_ ratio were considered ancillary outcomes. Moreover, we recorded adverse events as reported. The overall number of devices associated adverse events were recorded, “clinically significant” adverse events were categorized into bleeding, thrombotic and ischemic events as well as technical or cannulation associated events, respectively. “Bleeding” comprises events reported as “clinically significant”, “clinically relevant” or “major”, “thrombotic and ischemic events” comprise membrane or pump clotting, cannula thrombosis, intravascular thrombosis or embolism as well as cannulation associated limb ischemia. Air in circuit, circuit leakage, pump failure, device malfunction or cannula breakdown were categorized as “technical or cannulation associated”. If the same patient underwent more adverse events, all of them were counted.

For controlled trials, we assessed the duration of ventilation, length of stay (ICU, hospital), mortality (ICU, hospital, 60-day), health-related quality of life at 6 months after inclusion and adverse events. Carbon dioxide extraction capacity (“higher” versus “lower”) was categorized according to [15], where systems allowing blood flows > 500–600 mL/min and using gas exchange membranes exceeding a surface of 0.60 m^2^ were categorized as “higher extractors”.

### Search methods for identification of studies

We built a tailored search algorithm for each database, using intervention-related terms for the topic “extracorporeal CO_2_ removal”. The detailed search strategy is available in the Additional Information (Additional file 1: File C).

A medical information specialist (BW) conducted a comprehensive electronic search from 1st January 2000 to 2nd March, 2022. Databases consulted included: MEDLINE, EMBASE.com, Cochrane Central Register of Controlled Trials (CENTRAL), Scopus, LILACS, ClinicalTrials.gov and Web of Science Core Collection (SCI-EXPANDED, SSCI, AHCI, CPCI-S, CPCI-SSH, ESCI). We did not apply any language limitations.

### Data collection and analysis

#### Study selection

**Fig. 1:**
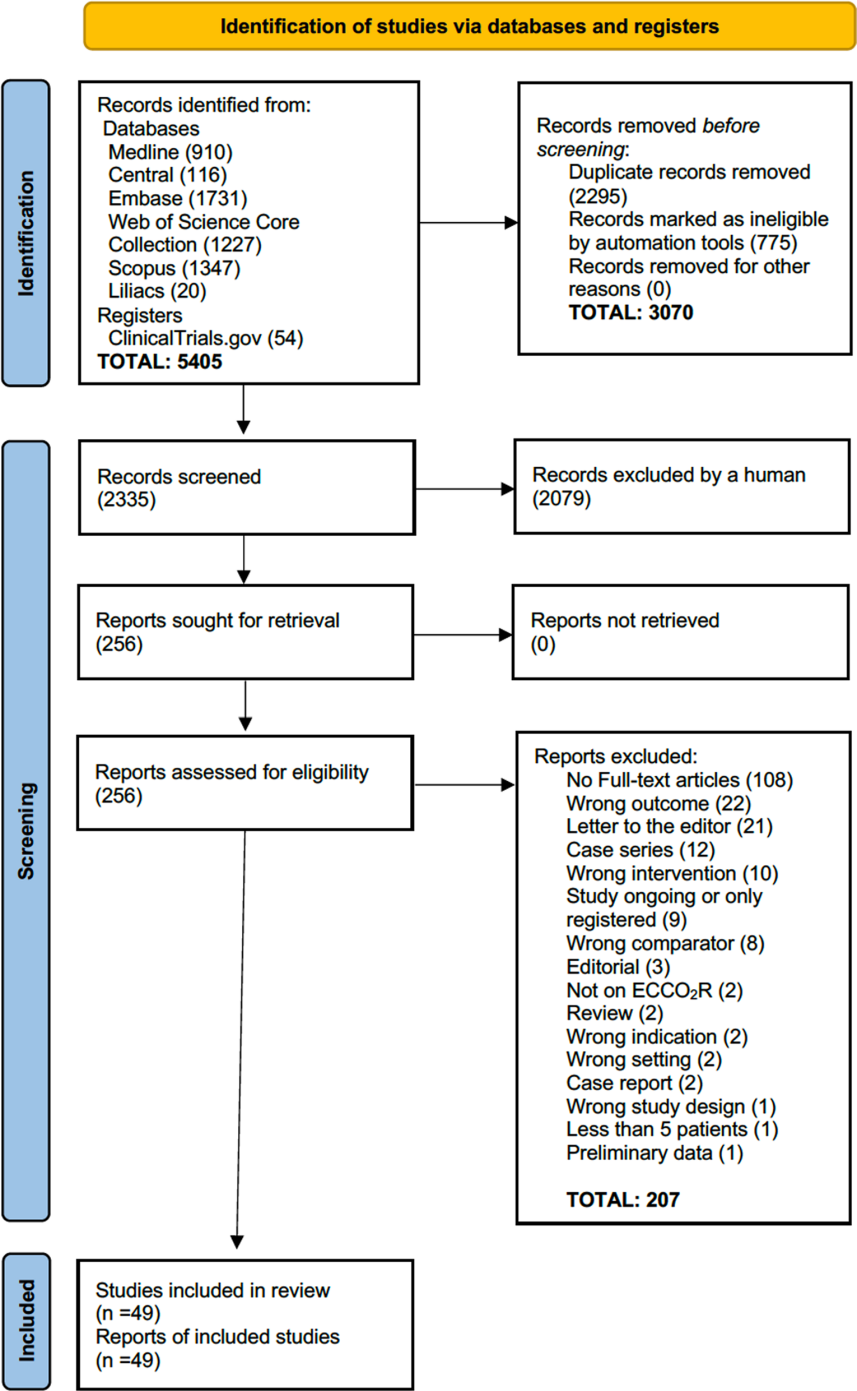
PRISMA flow diagram for new systematic reviews which included searches of databases and registers only

Using Covidence software (Covidence systematic review software, Veritas Health Innovation, Melbourne, Australia. Available at www.covidence.org), two independent reviewers (AS, CK) scrutinized the electronic search outcomes. The process of excluding non-relevant studies unfolded in stages, as outlined in Fig. 1.

Initially, studies not meeting the criteria were identified and excluded based on their titles and abstracts. Then, reviewers (AS, CK) independently reviewed the full texts of the remaining articles for relevance. Discrepancies in their selections were collaboratively resolved after each review phase.

### Data extraction and management

Two reviewers (AS, CK) independently extracted data on study design, setting, population, intervention, and outcomes using a pre-established form. We sought the most granular numerical data pivotal for our analyses. If crucial data was graphically represented, it was manually gleaned by the same two reviewers (AS, CK) without aid of any software. Discrepancies in data extraction were resolved through discussion, with a third party (TS, HH or AH) available for arbitration if required.

### Assessment of risk of bias in included studies

To assess bias risk in before-after comparisons we used the ROBINS-I tool [17] both in the intervention arm of the randomized trials and in the non-randomized studies. Two independent reviewers (AS, CK) conducted bias evaluation for each study. Disagreements were resolved through discussion, with a third party (TS, HH or AH) mediating when necessary.

### Measures of treatment effect

Within and across studies we used the mean of differences (intra-individual difference) as the measure of treatment effect. Where the studies lacked measures of variability for these intra-individual differences (change values) we used a range of correlation coefficients to calculate the appropriate standard deviation of change values as described in the Cochrane Handbook. Across the randomised controlled trials (RCT), we used the odds ratio for the assessment of mortality effects, and the mean difference between groups for effects on length of stay and ventilation days.

### Dealing with missing data

In instances of missing data, we contacted the corresponding authors for information, avoiding data imputation models. For isolated missing values like standard deviations, we replaced them with the average across the other studies. Data in the studies were presented as mean or medians, standard deviations, ranges, interquartile ranges, standard errors, or confidence intervals. Assuming normal distribution for outcomes, we converted medians to means and transformed interquartile ranges, standard errors, and confidence intervals into standard deviations using methods from the Cochrane Handbook.

### Assessment of heterogeneity

We assessed clinical heterogeneity based on clinical expert knowledge, and methodological heterogeneity by assessing study design details. We assessed statistical heterogeneity by inspecting forest plots and by calculating the I^2^ statistics, which we interpreted in the respective context.

### Data synthesis

In the absence of relevant clinical or methodological heterogeneity, we planned pooling the study outcomes. Given the nature of the intervention and populations we assumed some degree of underlying heterogeneity, therefore we used random effects models as default. Meta-analysis for primary outcomes was conducted using Stata's 'metan' routine (Stata Corp, College Station, TX) calculating pooled mean differences with 95% confidence intervals. We calculated pooled adverse event rates with 95% confidence intervals using multilevel mixed-effects Poisson regression. We avoided combining effects from different study designs, such as effects from before-after comparisons with effects from interventional parallel-group controlled trials, but we used the before-after effects from the intervention group from RCTs. RCTs outcomes, including mortality, ventilator-free days, length of stay, and adverse events were analysed using Stata’s ‘meta’ routine. We report our estimates of binary outcomes from RCTs as odds ratio with 95% confidence intervals. For outcomes with a low frequency, we calculated the Peto odds ratio instead. Subgroup analyses were pre-defined based on the underlying disease (ARDS, COPD, bridging to LTX), and technology used (lower versus higher extraction systems).

### Sensitivity analysis

Sensitivity analyses were conducted to evaluate the influence of bias risk on key outcomes, categorizing studies based on their ROBINS-I risk levels (low, moderate, serious, or critical).

## Results

After searching the databases, 5405 articles met our inclusion criteria for further screening (Fig. 1 and Additional file 1: File B and C). After removing duplicates and ineligible records with electronic tools, 2,079 papers were excluded by the screening team as irrelevant based on title and abstract. After a full text review of 256 studies, 207 were excluded, resulting in 49 studies for inclusion. These comprised three prospective open-label randomized studies, 18 prospective observational, and 28 retrospective observational studies, totalling 1,661 ECCO_2_R patients. The median number of patients per study was 20 (range 7–186). Additional file 1: Table S1 summarizes the main characteristics of the studies included. Notably, two studies reported separate cohorts for ARDS and COPD patients undergoing ECCO_2_R with distinct therapeutic goals [18, 19]. Since the results were reported separately without pooling, each cohort was treated as an individual study, leading to 51 datasets being analysed independently. Additional file 1: Table S1 marks two such trials as (a) and (b). In Augy et al.’s study [19], 70 ECCO_2_R patients with various indications were included, but parameters on gas exchange and ventilation for only ARDS and COPD patients (cohort a: n = 24, cohort b: n = 30) were analysed. Device characteristics used in ECCO_2_R are detailed in Additional file 1: Table S2.

Of the six ventilation parameters of interest (plateau pressure, PEEP, tidal volume, respiratory rate, PaCO_2_, PaO_2_/FiO_2_ ratio, and pH), only PaCO_2_ could be extracted from all studies, except for one [20]. Only 16 studies reported on all six parameters [4, 6, 18, 21–33]. Additional file 1: Table S3 shows the parameters available for each included study. Risk of bias assessment (Robins-I tool) revealed 4 studies with a low risk [31, 34–36], 14 with moderate risk [6, 8, 19, 30, 33, 37–44], and 30 with serious risk [4, 18, 21–29, 32, 45–60]. Three studies were categorized as critical risk [61–63] (Additional file 1: Figure S1).

### Overall data

Pooling all studies revealed a significant overall reduction in PaCO_2_ following ECCO_2_R initiation (Additional file 1: Figure S2a) although in 6 out of 50 studies, no decrease or even an increase in PaCO_2_ was observed [6, 25, 26, 29, 31, 58]. This included five studies with the primary goal of reducing tidal volume and concomitantly avoiding respiratory acidosis by ECCO_2_R. Tidal volume was reduced from 6 to 4 ml/kg (predicted body weight) in four studies [6, 25, 26, 29] and from 6.5 to 4.5 ml/kg (predicted body weight) in one study [31]. In another study on patients suffering from coronavirus disease 2019 (COVID-19) ARDS with hypercapnia, ECCO_2_R was not able to reduce PaCO_2_ significantly [58]. Concomitantly with overall decrease of PaCO_2_, pH increased significantly (Additional file 1: Figure S2b).

Oxygenation, expressed by PaO_2_/FiO_2_ ratio, increased significantly overall. There were 18 studies out of 37 however, which did not observe a significant increase (Additional file 1: Figure S3a). PEEP levels remained grossly unchanged (Additional file 1: Figure S3b). Significant reductions were seen in both plateau pressure and tidal volume, with exception in 6/27 and 10/29 studies, respectively (Additional file 1: Figures S4a and 4b). A significant reduction of respiratory rate could be observed overall (except for 8/30 studies) (Additional file 1: Figure S4c).

### Diagnoses subgroups

**Fig. 2:**
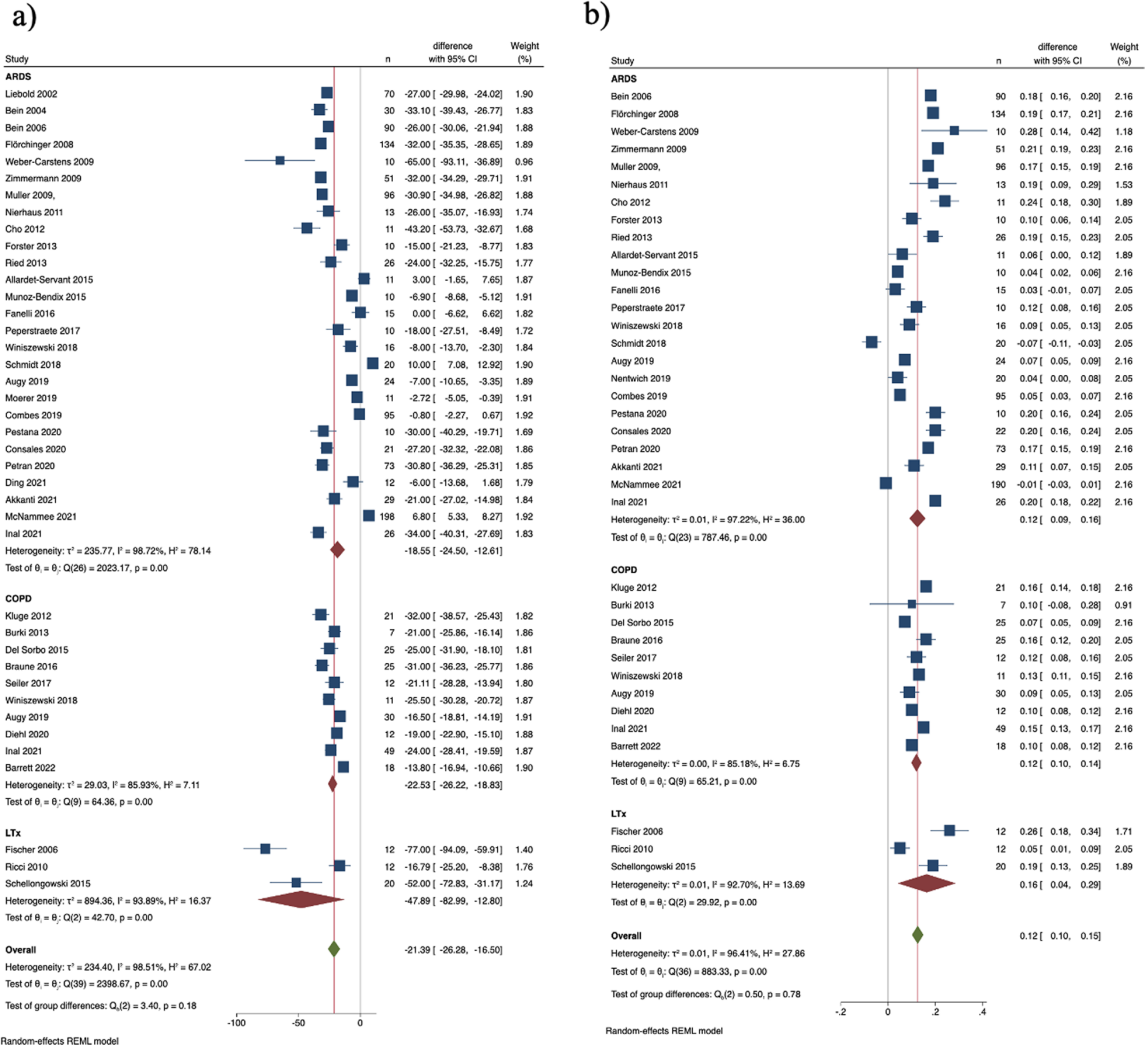
Change of (a) PaCO_2_, mmHg, and (b) pH within 24 h after initiating ECCO_2_R (diagnoses subgroups)

Table 1 outlines diagnoses and main ECCO_2_R therapy targets. Across ARDS, COPD and LTX subgroups, PaCO_2_ significantly decreased, and pH significantly increased (Fig. 2a, b). PEEP levels remained unchanged in all three subgroups (Additional file 1: Figure S5b).

**Table 1:** Summary of studies included for analysis (for details on each study refer to Additional file 1: Table S1)

Diagnosis subgroup	Patients (n) on ECCO_2_R	Device(s) (number of studies)	Type of study (number of studies)	Primary clinical goals (number of studies)
ARDS	1179 in 27 studies	Higher extraction systems:	Cohort, retrospective (11)	Improve gas exchange (4)
		AV ILA® (16)	Cohort, prospective (11)	More protective ventilation/reduction of tidal volume (10)
		ILA Activve® (2)	Randomized prospective trial (2)	Improve gas exchange + more protective ventilation (10)
		Cardiohelp HLS 5.0® (2)		Safety, effects on pH, ventilator settings, and hemodynamics (1)
		Lower extraction systems:		Reduction of PaCO_2_ and ICP (1)
		RRT + ECCO_2_R (2)		More protective ventilation, facilitate weaning, avoid intubation (1)
		Prismalung® (5)		
		Hemolung RAS® (6)		
		Abylcap® (1)		
		EQUA-smart® (1)		
Bridge to LTX	44 in 3 studies	Higher extraction systems:	Cohort, retrospective (3)	Improve gas exchange (2)
		AV ILA® (3)		Improve gas exchange + more protective ventilation (1)
		ILA Activve® (1)		
		Lower extraction systems:		
		Decap Smart® (1)		
AECOPD	140 in 8 studies	Higher extraction systems:	Cohort, retrospective (2)	Avoid intubation (6)
		AV ILA® (2)	Cohort, prospective (5)	Facilitate weaning (1)
		ILA Activve® (2)	Randomized open-label prospective trial (1)	Reduction of PaCO_2_ (1)
		Cardiohelp HLS 5.0® (1)		
		Lower extraction systems:		
		Hemolung RAS® (5)		
		Decap Smart® (1)		
		Prismalung® (1)		
Mixed	298 in 12 studies	Higher extraction systems:	Cohort, retrospective (10)	Improve gas exchange (7)
		AV ILA® (3)	Cohort, prospective (2)	Improve gas exchange + more protective ventilation (2)
		ILA Activve® (1)		Avoid intubation (2)
		Homburg Lung (1)		More protective ventilation (3)
		Lower extraction systems:		
		Hemolung RAS® (3)		
		RRT + ECCO_2_R (1)		
		ProLung® (2)		
		Decap Smart® (1)		
		Prismalung® (1)		

ECCO_2_R, Extracorporeal carbon dioxide removal; ARDS, acute respiratory distress syndrome; COPD, chronic obstructive pulmonary disease; AECOPD, acute exacerbated chronic obstructive pulmonary disease; LTX, lung transplantation; RRT, Renal replacement therapy; ICP, Intracranial pressure

#### ARDS

**Fig. 3:**
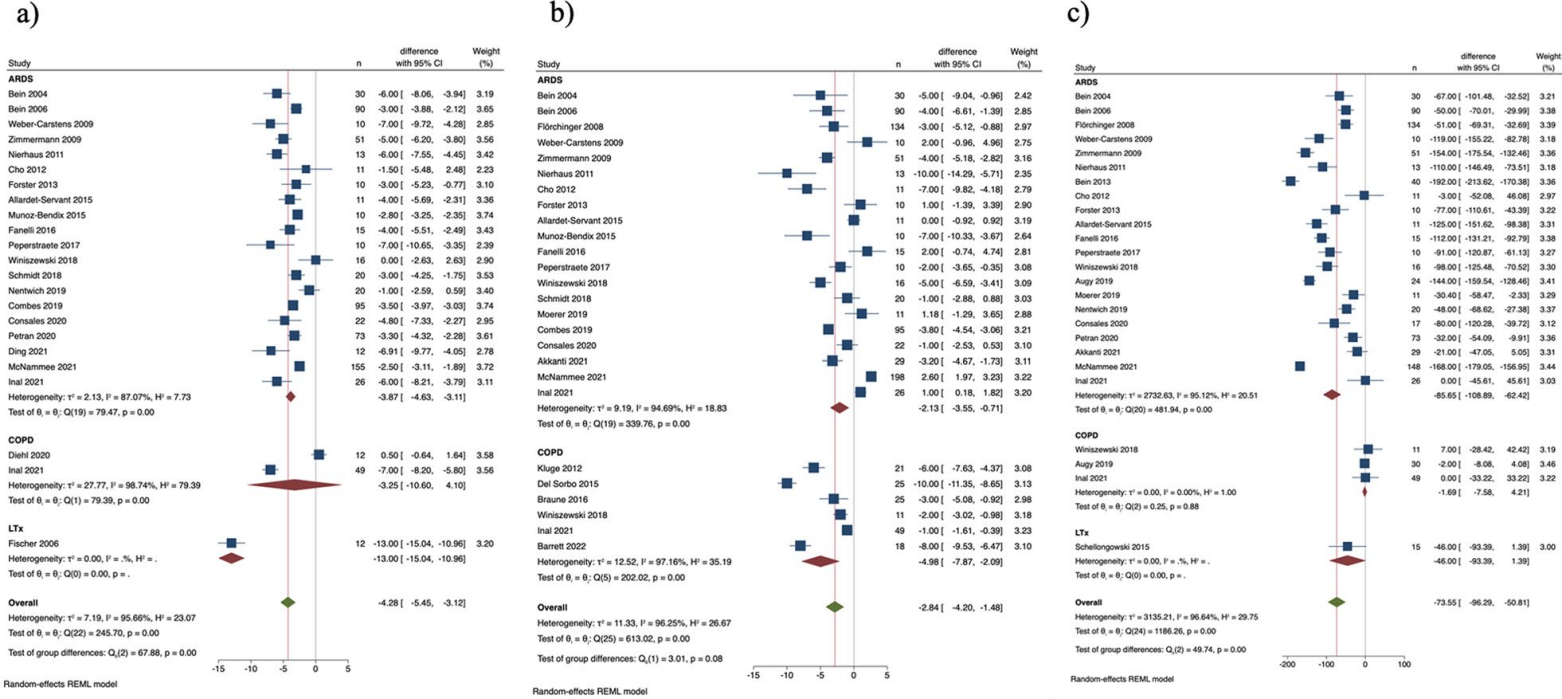
Change of (a) plateau pressure, cmH_2_O, (b) respiratory rate, breaths/min, and (c) tidal volume, mL within 24 h after initiating ECCO_2_R (diagnoses subgroups)

PaCO_2_ decreased and pH increased (Fig. 2a, b) in ARDS patients. PaO_2_/FiO_2_ ratio increased significantly (Additional file 1: Figure S5a), and plateau pressure and respiratory rate decreased (Fig. 3a, b). Tidal volume reduction was statistically significant only in ARDS patients (Fig. 3c).

#### AECOPD

PaCO_2_ decreased and pH increased to a statistically significant extent (Fig. 2a, b). PaO_2_/FiO_2_ ratio did not increase to a statistically significant extent (Additional file 1: Figure S5a) while respiratory rate decreased significantly (Fig. 3b). Tidal volume did not change to significant extent (Fig. 3c). Of note, only 2 studies reported on plateau pressure in COPD patients, which did not decrease significantly (Fig. 3a).

#### Bridge to LTX

PaCO_2_ decreased and pH increased as in ARDS and AECOPD patients (Fig. 2a, b). PaO_2_/FiO_2_ ratio increased significantly (Additional file 1: Figure S5a). Plateau pressure significantly decreased (Fig. 3a), while no study reported on significant changes in respiratory rate (Fig. 3b). Only one study reported on plateau pressure changes. Tidal volume did not change to significant extent (Fig. 3c).

### Lower extraction versus higher extraction

**Fig. 4:**
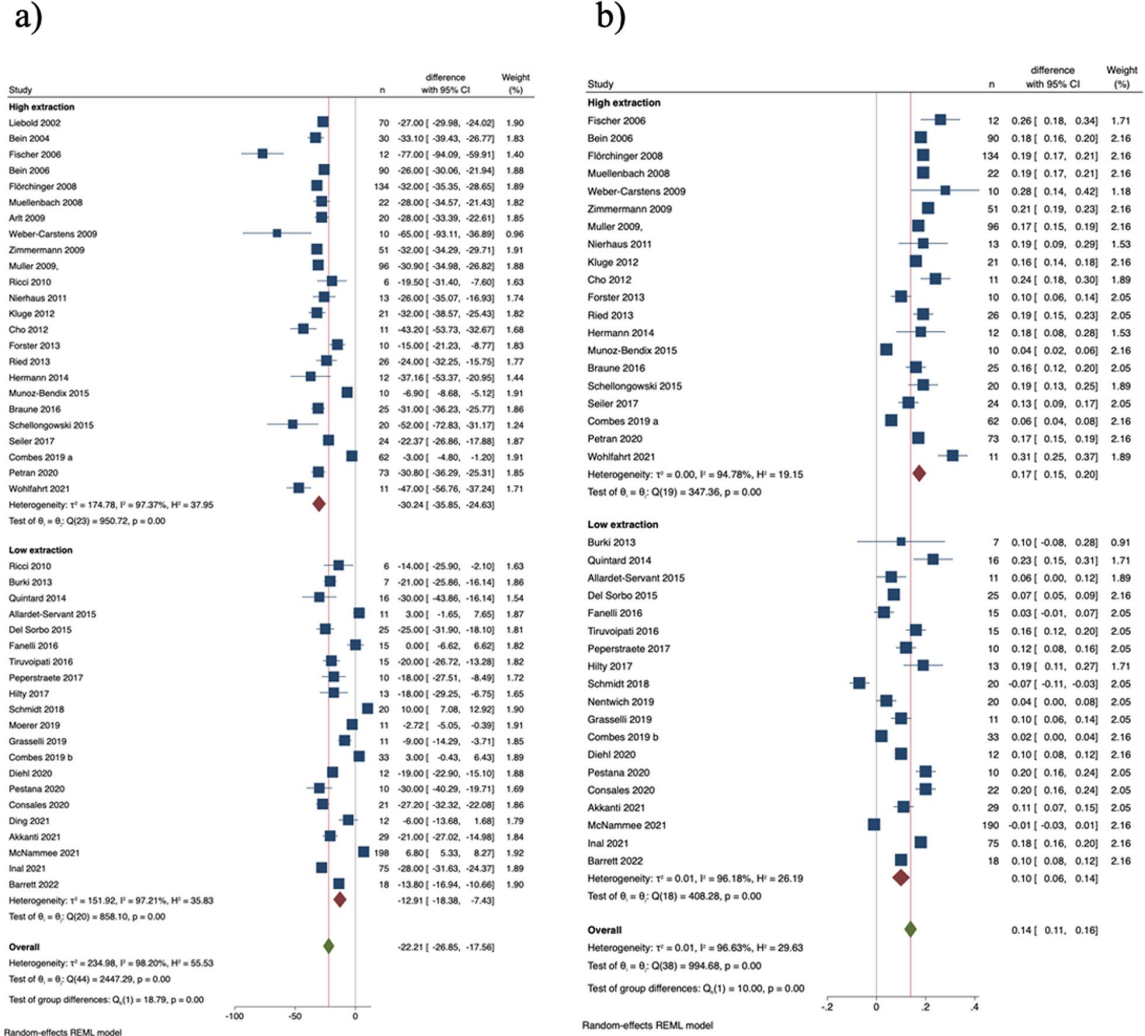
Change of (a) PaCO_2_, mmHg, and (b) pH within 24 h after initiating ECCO_2_R (lower extraction and higher extraction subgroups)

Higher extraction ECCO_2_R devices were used in 22 studies and lower extraction devices in another 22, with five studies (seven datasets) using both types (Table 1). In both subgroups, PaCO_2_ was significantly reduced, more so with higher extraction devices (Fig. 4a). For ARDS patients, the lowest PaCO_2_ decrease was seen with lower extraction devices (Additional file 1: Figure S6a). A similar trend was observed in pH increase (Fig. 4b). PaO_2_/FiO_2_ ratio increased significantly in both subgroups, while PEEP remained unchanged (Additional file 1: Figure S7a and b). However, in COPD/LTX patients, the PaO_2_/FiO_2_ ratio did not significantly increase in either extraction subgroup (Additional file 1: Figure S8a). In both higher and lower extraction subgroups, plateau pressure and tidal volume decreased significantly (Additional file 1: Figure S9a and b). However, in COPD and LTX patients, no significant reduction in plateau pressure was observed with lower extraction devices (Additional file 1: Figure S10a). Tidal volume reduction was significant in ARDS patients for both extraction subgroups, but not in COPD/LTX patients (Additional file 1: Figure S10b). The respiratory rate significantly declined in both extraction subgroups, notably more in the higher extraction group In ARDS patients, the use of lower extraction devices did not lead to a significant reduction in respiratory rate (Additional file 1: Figure S10c).

### Effects from randomized controlled trials (RCTs)

We identified three RCTs: two with mild to moderate ARDS patients [20, 31]. And one with AECOPD patients on NIV [36]. The aim of the ARDS trials was to reduce the invasiveness of ventilation to an “ultraprotective level” in the ECCO_2_R arms, while standard “protective” ventilation was used in the control arms. In the AECOPD study, patients on NIV with high risk of failure were randomized to NIV plus ECCO_2_R or continued NIV only.

All studies reported on mortality and length of hospital/ICU stay, but only the ARDS studies mentioned ventilation duration. None assessed health-related quality of life. No significant differences in mortality or ventilator-free days at day 28 (VFD-28), were observed between ECCO_2_R and control groups, but ECCO_2_R groups had longer ICU and hospital stays (Table 2).

**Table 2:** Randomized controlled trials

Trial	Bein 2013	McNamee 2021	Barrett 2022	Pooled effect size [95% confidence interval]
Patients (n)	ECCO_2_R = 40 Control = 39	ECCO_2_R = 200 Control = 205	ECCO_2_R = 9 Control = 9	
Diagnosis/Indication	ARDS	ARDS	AECOPD	
Mortality	Hospital: 17.5% versus 15.4%; *p* = 1.000	90-day: 41.5% versus 39.5%: * p* = 0.68	Hospital: 34% versus 11%; * p* = 0.58	OR 0.89 [0.62, 1.29]
Length of stay (days)	ICU: 31.3 ± 23 versus 22.9 ± 11; * p* = 0.144 Hospital: 46.7 ± 33 versus 35.1 ± 17; * p* = 0.113	ICU: 14 (7 to 26) versus 13 (7 to 22); * p* = 0.67 Hospital: 22 (8 to 39) versus 18 (9 to 35); * p* = 0.65	ICU: 6.7 (5.5–7.3) versus 1.9 (1.7–2.2) h; * p* = 0.001 Hospital: 10 (9.2–14.0) versus 5.2 (4.3–8.9); * p* = 0.014	Days at ICU: 3.78 [0.40, 7.17] Days in Hospital: 4.82 [2.33, 7.32]
Ventilator free days (VFD)	VFD 28: 10.0 ± 8 versus 9.3 ± 9; * p* = 0.779	VFD 28: 7.1 (8.8) versus 9.2 (9.3); * p* = 0.02	Not reported	VFD-28: − 1.21 [− 3.77, 1.35]
Quality of Life at 6 months	Not reported	Not reported	Not reported	
Adverse events (PetoOR [95% confidence interval])				*PetoOR*
any AE	Not reported	11.18 [4.67, 26.76]	15.55 [0.70, 346]	11.46 [4.95, 26.54]
Bleeding	Not reported	5.58 [2.93, 10.63]	9.65 [0.87, 107]	5.79 [3.11, 10.78]
Thrombosis	Not reported	4.01 [0.80, 20.09]	Not reported	4.01 [0.80, 20.09]
Technical	Not reported	8.01 [2.14, 29.96]	7.39 [0.15, 372]	7.94 [2.27, 27.74]

### Adverse events

All studies but three [40, 58, 62] reported on device associated adverse events, involving 1551 patients. Overall, the adverse event rate was 19% (95% CI 12–28). Rates of bleeding events, thrombotic or ischaemic events, and technical adverse events were 5%, 7% and 2%, respectively (Table 3 and Additional file 1: Table S4). A number of studies reported on adverse events aside these categories like haemolysis or thrombocytopenia, in most cases not affecting therapy. Haemolysis was reported in 42 patients, in 38 cases associated with the Hemolung RAS^©^ system. Thrombocytopenia was reported in three studies only affecting 17 patients. Lower limb ischemia was reported in 34/658 patients leading to compartment syndrome in 10 and amputation in 3 patients, all of them associated with arterial cannulation using the pumpless arterio-venous ILA^©^ system. These events were categorized as “thrombotic or ischemic”. Eleven cases of intracerebral haemorrhage were reported, categorized as “bleeding”. Among RCTs the overall adverse event rate, bleeding and technical adverse events were significantly higher than in the intervention group compared to controls (Table 2).

### Risk of bias

Among the studies analysed, only 4 were categorized as having a low risk of bias (Additional file 1: Figure S1). Subgroup analysis based on the Robins-I category showed minimal bias impact on the parameters studied (Additional file 1: Figure S11a–g). Overall, before-after studies demonstrated a robust effect on CO_2_ removal and related parameters.

## Discussion

ECCO_2_R, using specifically designed devices has been in use for about two decades, with a variety of devices introduced for different clinical indications and therapeutic goals. However, no systematic analysis of the effects of ECCO_2_R has yet pooled data from studies across all devices and indications.

Our main finding is that the primary goal of ECCO_2_R, i.e., elimination of carbon dioxide, reduction of PaCO_2_, and acidosis can be achieved irrespective of device and indication. Devices designed for higher extraction and those for lower extraction both produce similar effects, though higher extraction devices do so more markedly. In ARDS patients, higher extraction devices more efficiently reduce PaCO_2_, tidal volume, and respiratory rate, while in COPD/LTX patients, they more effectively lower plateau pressure.

A retrospective subgroup analysis of data from a prospective cohort study on ARDS patients indicated that “ultraprotective” ventilation was more commonly achieved using devices with higher CO_2_ extraction capacity [15]. “Higher extraction” is not a well-defined term, but instead refers to ECCO_2_R systems with larger gas exchange membrane surface operating at blood flows over 600 mL/min. Additional file 1: Table S2 indicates that “lower extraction” devices operate at blood flows below 500 mL/min and are mainly based on CRRT technology. The Hemolung RAS system falls in between, operating around 500 mL/min, with a relatively small membrane surface of 0,59 m^2^. In agreement with Combes et al. [15] we thus categorized the Hemolung RAS system as “lower extractor”. Higher extraction systems typically operate above 800 mL/min of blood flow and utilize larger membranes. Our results suggests that in scenarios such as spontaneously breathing patients (e.g., in AECOPD or during bridging to LTX to avoid mechanical ventilation) or in individuals with a very high carbon dioxide burden, lower extraction devices may not be adequate to achieve therapeutic goals. Lower extraction devices are promoted as easier to use and less invasive, yet there is no proven reduction in side effects such as bleeding. Notably, bleeding rates were high with the Hemolung RAS system [15, 31], potentially due to hemolysis induced by the centrifugal pump, which appears to increase at low blood flow rates [64].

Despite the proven beneficial effects on gas exchange and mechanical ventilation, evidence remains debatable. The concept of “ultraprotective” ventilation in ARDS patients enabled by ECCO_2_R, although shown to reduce biotrauma [65], has failed to improve clinical outcomes [20, 31]. In the light of more favourable evidence with respect to VV ECMO in severe ARDS [66, 67], the role of ECCO_2_R in ventilating below standard protective settings remains questionable. When pooling the so far published three randomized prospective trials, we could not detect any effect on mortality (Table 2). The trials, however, are heterogeneous: The Xtravent Study on ARDS patients was stopped prematurely and thus underpowered but showed at least a positive effect on duration of ventilation in the subgroup with more severe ARDS [20]. The large and well-conducted REST Trial demonstrated no mortality benefits from ECCO_2_R and revealed a negative impact on ventilation duration [31]. Adverse events related to ECCO_2_R were frequently recorded. The third RCT involved a small cohort of eighteen spontaneously breathing patients with AECOPD undergoing NIV with a high risk of failure. ECCO_2_R improved physiological parameters and reduced the duration of NIV. However, no mortality benefits were observed. Despite varied ECCO_2_R indications and study designs, all studies reported on a longer hospital stay in ECCO_2_R patients, a finding that became significant upon data pooling (Table 2). This increased hospitalization may stem from different clinical management of ECCO_2_R patients in open-label studies [36], and a higher incidence of adverse events such as bleeding [31].

Overall, the reported adverse events rate was as high as 19%. This number has to be regarded with caution as adverse events were defined heterogeneously and often assessed from retrospective studies. Severity of adverse events was not categorized according to standard criteria in most studies. The reported adverse events rate ranged between zero to 77%, pointing towards a considerable heterogeneity between studies concerning definition and documentation of adverse events. Our data however underline that ECCO_2_R can lead to severe adverse events, many of them coagulation associated like major bleeding or thrombotic events. While for bleeding, we detected no major difference between higher and lower extraction systems, rate of thrombotic or ischemic events occurred more often in patients treated by higher extractions systems. It has to be taken into account however, that a part of the ischemic events were specifically due to arterial cannulation using a pumpless system. The only large, prospective, randomized trial assessing outcome and complications in 412 patients with acute hypoxemic respiratory failure [31] found no mortality benefit, and a high rate of serious adverse events associated with ECCO_2_R. These results indicate that ECCO_2_R is not appropriate for broad clinical adoption in ARDS and should be used with extreme caution, most likely in the setting of rigorously designed research protocols.

It seems that hypercapnic lung failure represents a more rewarding indication for ECCO_2_R, which has been shown to be a useful tool to prevent mechanical ventilation in patients suffering from AECOPD and failure of NIV [34, 35, 41], as well as a therapeutic option to bridge patients with terminal hypercapnic lung failure to LTX [8, 9]. ECCO_2_R has also been reported as successful therapy in refractory status asthmaticus. Only case reports on the use of low-flow ECCO_2_R systems have been published so far. There are however retrospective studies on the use of ECMO [68–70]. VV ECMO with the primary goal of treating hypercapnia has been shown to be a very successful option for refractory asthma [70]. As high-flow extracorporeal gas exchange systems were not within the scope of our review and ECMO settings were not reported in most of these studies, we chose to exclude this indication from our analysis. Our findings however suggest that in hypercapnic patients suffering from AECOPD or during bridging to LTX, higher extraction devices may be superior regarding their effects on plateau pressure and especially the more pronounced effect on respiratory rate, which could contribute to a reduction of overinflation in patients with obstructive lung diseases. Again, high-quality evidence supporting the effectiveness of ECCO_2_R in treating hypercapnic lung failure remains lacking, thus classifying it as experimental therapy.

Our findings are subject to several limitations. First, the therapeutic goals of ECCO_2_R throughout the trials included were heterogeneous: Some studies included hypercapnic patients with the goal to reduce acidosis, while others set out to reduce tidal volume and ventilation pressures enabled by ECCO_2_R. Studies on AECOPD and/or bridging to LTX aimed for avoiding mechanical ventilation or assisting weaning. As one might expect, the effect on CO_2_ levels was more pronounced in studies including hypercapnic patients. Interestingly though, when pooling all available data, the effects were quite homogenously directed in the same direction. Moreover, not all studies reported on all data (Additional file 1: Table S3). In spontaneously breathing patients, ventilatory settings were not reported and changes in respiratory rate were either dependent on the patients themselves (if breathing spontaneously) or on the ventilation protocol applied. Second, we found a considerable risk of bias in most of the studies included in our work. Only four studies were categorized as yielding a low risk of bias. When analysing the data according to risk of bias category, however, results were quite uniform in each category.

In summary, we found a robust effect of ECCO_2_R on CO_2_ removal and related parameters. Data from three RCTs, however, did not indicate a significant mortality benefit. Additionally, ECCO_2_R was associated with a high rate of serious adverse events. Based on existing evidence, ECCO_2_R cannot be recommended for ARDS outside of clinical trials. While it may show greater effectiveness in hypercapnic lung failure, it remains experimental.

### Supplementary Information

**Additional file 1. Additional File A**. PRISMA 2020 checklist. **Additional File B**. Criteria for considering studies for this review. **Additional File C**. Search strategy. **Additional Table S1**. Studies. **Additional Table S2**. Devices designed for ECCO2R and their basic specifications. **Additional Table S3**. Available data from included studies. **Additional Table S4**. Adverse Events from observational studies. **Additional Figure S1**: Risk of bias assessment (Robins-I tool). **Additional Figure S2** a, and b: Change of (a) PaCO2, mmHg and (b) pH within 24 hours after initiating ECCO2R (all studies). **Additional Figure S3** a, and b: Change of (a) PaO2/FiO2 ratio, mmHg and (b) PEEP, cmH2O within 24 hours after initiating ECCO2R (all studies). **Additional Figure S4** a, b, and c: Change of (a) plateau pressure, cmH2O, (b) tidal volume, mL, and (c) respiratory rate, breaths/min within 24 hours after initiating ECCO2R (all studies). **Additional Figure S5** a, and b: Change of (a) PaO2/FiO2 ratio, mmHg and (b) PEEP, cmH2Owithin 24 hours after initiating ECCO2R (diagnoses subgroups). **Additional Figure S6** a, and b: Change of (a) PaCO2, mmHg (a) and (b) PaO2/FiO2 ratio, mmHg within 24 hours after initiating ECCO2R according to diagnosis and extraction. **Additional Figure S7** a, and b: Change of (a) PaO2/FiO2 ratio, mmHg and (b) PEEP, cmH2O within 24 hours after initiating ECCO2R (lower extraction and higher extraction subgroups). **Additional Figure S8** a, and b: Change of (a) PaO2/FiO2 ratio, mmHg and (b) PEEP, cmH2O within 24 hours after initiating ECCO2R according to diagnosis and extraction. **Additional Figure S9** a, and b: Change of (a) plateau pressure, cmH2O and (b) tidal volume, mL within 24 hours after initiating ECCO2R (lower extraction and higher extraction subgroups). **Additional Figure S10** a, b, and c: Change of (a) plateau pressure, cmH2O, (b) tidal volume, mL, and (c) respiratory rate, breaths/min within 24 hours after initiating ECCO2R according to diagnosis and extraction. **Additional Figure S11** a-g: (a) PaCO2, mmHg, (b) pH, (c) PaO2/FiO2 ratio, mmHg and (d) PEEP, cmH2O, (e) plateau pressure, cmH2O, (f) tidal volume, mL and (g) respiratory rate, breaths/min according to risk of bias.

## Data Availability

The datasets generated and/or analysed during the current study are available in this article and its additional files.

## Abbreviations

AECOPD: Acute exacerbated chronic obstructive pulmonary disease
AKI: Acute kidney injury
ARDS: Acute respiratory distress syndrome
CARDS: COVID-19 ARDS
COPD: Chronic obstructive pulmonary disease
COVID-19: Coronavirus disease 2019
CRRT: Continuous renal replacement therapy
ECCO_2_R: Extracorporeal carbon dioxide removal
ECMO: Extracorporeal membrane oxygenation
HE: Higher extraction system
ICU: Intensive care unit
ILA®: Interventional Lung Assist®
LE: Lower extraction system
LTX: Lung transplantation
NIV: Non-invasive ventilation
PEEP: Positive end-expiratory pressure
RCT: Randomized controlled trial
RRT: Renal replacement therapy
VFD: Ventilator free days
