# Supplementary material for: Effects of extracorporeal CO2 removal on gas exchange and ventilator settings: a systematic review and meta-analysis
Source: Crit Care. 2024 Apr 30;28:146. doi: 10.1186/s13054-024-04927-x (PMC11061932; doi:10.1186/s13054-024-04927-x)

**Effects of extracorporeal CO_2_ removal on gas exchange and ventilator settings: A systematic review and meta-analysis**

**Additional files**

Alexandra-Maria Stommel^1^, Harald Herkner^1^*, Calvin Lukas Kienbacher^1^_,_ Brigitte Wildner^2^, Alexander Hermann^3^, Thomas Staudinger^3^

^1^Department of Emergency Medicine, Medical University of Vienna, Waehringer Guertel 18-20, 1090 Vienna, Austria

^2^University Library, Medical University of Vienna, Waehringer Guertel 18-20, 1090 Vienna, Austria

^3^Department of Medicine I, Intensive Care Unit 13i2, Medical University of Vienna, Waehringer Guertel 18-20, 1090 Vienna, Austria

***Correspondence:**

**Harald Herkner**

Department of Emergency Medicine

Medical University of Vienna, Austria

Waehringer Guertel 18-20, 1090 Vienna, Austria

Phone: +43 40400 19540

mail: harald.herkner@meduniwien.ac.at

**Additional File A.** PRISMA 2020 checklist.

| **Section and Topic** | **Item #** | **Checklist item** | **Location where item is reported** |
| --- | --- | --- | --- |
| **TITLE** | | |  |
| Title | 1 | Identify the report as a systematic review. | Title page |
| **ABSTRACT** | | |  |
| Abstract | 2 | See the PRISMA 2020 for Abstracts checklist. | p.1 |
| **INTRODUCTION** | | |  |
| Rationale | 3 | Describe the rationale for the review in the context of existing knowledge. | pp. 2-3 |
| Objectives | 4 | Provide an explicit statement of the objective(s) or question(s) the review addresses. | p.3 |
| **METHODS** | | |  |
| Eligibility criteria | 5 | Specify the inclusion and exclusion criteria for the review and how studies were grouped for the syntheses. | pp. 3-5 |
| Information sources | 6 | Specify all databases, registers, websites, organisations, reference lists and other sources searched or consulted to identify studies. Specify the date when each source was last searched or consulted. | p.5, Ad. F. pp3-5 |
| Search strategy | 7 | Present the full search strategies for all databases, registers and websites, including any filters and limits used. | p.5, Ad. F. p. 5 |
| Selection process | 8 | Specify the methods used to decide whether a study met the inclusion criteria of the review, including how many reviewers screened each record and each report retrieved, whether they worked independently, and if applicable, details of automation tools used in the process. | pp. 5-7 |
| Data collection process | 9 | Specify the methods used to collect data from reports, including how many reviewers collected data from each report, whether they worked independently, any processes for obtaining or confirming data from study investigators, and if applicable, details of automation tools used in the process. | pp. 5-7 |
| Data items | 10a | List and define all outcomes for which data were sought. Specify whether all results that were compatible with each outcome domain in each study were sought (e.g. for all measures, time points, analyses), and if not, the methods used to decide which results to collect. | pp. 7-8, Table S2 |
|  | 10b | List and define all other variables for which data were sought (e.g. participant and intervention characteristics, funding sources). Describe any assumptions made about any missing or unclear information. | pp. 7-8 |
| Study risk of bias assessment | 11 | Specify the methods used to assess risk of bias in the included studies, including details of the tool(s) used, how many reviewers assessed each study and whether they worked independently, and if applicable, details of automation tools used in the process. | p. 6 |
| Effect measures | 12 | Specify for each outcome the effect measure(s) (e.g. risk ratio, mean difference) used in the synthesis or presentation of results. | p.6 |
| Synthesis methods | 13a | Describe the processes used to decide which studies were eligible for each synthesis (e.g. tabulating the study intervention characteristics and comparing against the planned groups for each synthesis (item #5)). | pp. 5-8  Table 1, Ad. Table S2 |
|  | 13b | Describe any methods required to prepare the data for presentation or synthesis, such as handling of missing summary statistics, or data conversions. | pp. 5-8 |
|  | 13c | Describe any methods used to tabulate or visually display results of individual studies and syntheses. | pp 5-8 |
|  | 13d | Describe any methods used to synthesize results and provide a rationale for the choice(s). If meta-analysis was performed, describe the model(s), method(s) to identify the presence and extent of statistical heterogeneity, and software package(s) used. | pp. 7-8 |
|  | 13e | Describe any methods used to explore possible causes of heterogeneity among study results (e.g. subgroup analysis, meta-regression). | pp. 7-8 |
|  | 13f | Describe any sensitivity analyses conducted to assess robustness of the synthesized results. | p.8 |
| Reporting bias assessment | 14 | Describe any methods used to assess risk of bias due to missing results in a synthesis (arising from reporting biases). | pp. 7-8 |
| Certainty assessment | 15 | Describe any methods used to assess certainty (or confidence) in the body of evidence for an outcome. | p. 8 |
| **RESULTS** | | |  |
| Study selection | 16a | Describe the results of the search and selection process, from the number of records identified in the search to the number of studies included in the review, ideally using a flow diagram. | p. 8, Figure 1 |
|  | 16b | Cite studies that might appear to meet the inclusion criteria, but which were excluded, and explain why they were excluded. | Figure 1 |
| Study characteristics | 17 | Cite each included study and present its characteristics. | Table 1 |
| Risk of bias in studies | 18 | Present assessments of risk of bias for each included study. | Ad. Figure S1 |
| Results of individual studies | 19 | For all outcomes, present, for each study: (a) summary statistics for each group (where appropriate) and (b) an effect estimate and its precision (e.g. confidence/credible interval), ideally using structured tables or plots. | Table 2, Figure 2-4, Ad. Fig. S2-11 |
| Results of syntheses | 20a | For each synthesis, briefly summarise the characteristics and risk of bias among contributing studies. | p.12 |
|  | 20b | Present results of all statistical syntheses conducted. If meta-analysis was done, present for each the summary estimate and its precision (e.g. confidence/credible interval) and measures of statistical heterogeneity. If comparing groups, describe the direction of the effect. | p.8-12 |
|  | 20c | Present results of all investigations of possible causes of heterogeneity among study results. | p. 8-12 |
|  | 20d | Present results of all sensitivity analyses conducted to assess the robustness of the synthesized results. | p. 8-12 |
| Reporting biases | 21 | Present assessments of risk of bias due to missing results (arising from reporting biases) for each synthesis assessed. | Ad. Fig. S1 |
| Certainty of evidence | 22 | Present assessments of certainty (or confidence) in the body of evidence for each outcome assessed. | Table 2, Fig 2-4, Ad. Fig. |
| **DISCUSSION** | | |  |
| Discussion | 23a | Provide a general interpretation of the results in the context of other evidence. | p.12-16 |
|  | 23b | Discuss any limitations of the evidence included in the review. | p.12-16 |
|  | 23c | Discuss any limitations of the review processes used. | p.12-16 |
|  | 23d | Discuss implications of the results for practice, policy, and future research. | p.12-16 |
| **OTHER INFORMATION** | | |  |
| Registration and protocol | 24a | Provide registration information for the review, including register name and registration number, or state that the review was not registered. | p.3 |
|  | 24b | Indicate where the review protocol can be accessed, or state that a protocol was not prepared. | p.3 |
|  | 24c | Describe and explain any amendments to information provided at registration or in the protocol. | p.3 |
| Support | 25 | Describe sources of financial or non-financial support for the review, and the role of the funders or sponsors in the review. | p.19 |
| Competing interests | 26 | Declare any competing interests of review authors. | p.19 |
| Availability of data, code and other materials | 27 | Report which of the following are publicly available and where they can be found: template data collection forms; data extracted from included studies; data used for all analyses; analytic code; any other materials used in the review. | p.19 |

*From:* Page MJ, McKenzie JE, Bossuyt PM, Boutron I, Hoffmann TC, Mulrow CD, et al. The PRISMA 2020 statement: an updated guideline for reporting systematic reviews. BMJ 2021;372:n71. doi: 10.1136/bmj.n71 For more information, visit: <http://www.prisma-statement.org/>

**Additional File B.** Criteria for considering studies for this review

**Types of studies**

Randomised controlled trials and observational studies with at least one before-after ECCO_2_R implantation comparison. We did not include case series (n<10) or case reports. We included studies of ECCO_2_R used for the treatment of ARDS, obstructive lung diseases, and as a bridge to lung transplantation. We did not include studies before the year 2000. No language restrictions were imposed.

**Types of participants**

Individuals in respiratory failure with invasive or non-invasive ventilation for acute respiratory failure undergoing ECCO_2_R in the setting of critical or emergency care. We included studies on adult patients, aged 18 years and over.

**Types of interventions**

Contemporary ECCO_2_R. The intervention is any Extracorporeal CO_2_ removal, i.e., a device in which the primary aim is CO_2_ removal. Devices used with a primary intent of oxygenation were excluded.

**Types of outcome measures**

*Primary outcomes*

Changes of gas exchange and ventilator setting within 24 hours after initiation of ECCO_2_R.

*Important outcomes*

Ventilator settings:

- Peak inspiratory or plateau pressure (Pmax)
- Tidal volume (VT)

Gas exchange:

- PaCO_2_
- pH

*Ancillary outcomes*

Ventilator settings:

- Positive end-expiratory pressure (PEEP)
- Respiratory rate (RR)

Gas exchange

- P/F-Ratio

According to a consensus on core outcome sets in interventional trials on ventilation, we screened literature for the following parameters:

- Duration of mechanical ventilation (time to first extubation, follow-up for 60 days for possible reintubation)
- Duration of stay (ICU, hospital)
- Mortality (ICU, hospital, 60 days)
- Health-related quality of life 6 months after inclusion

***Search methods for identification of studies***

**Search terms**

We developed a search syntax appropriate for the respective databases. The search included at least the following terms for the intervention: “extracorporeal carbon dioxide removal” OR

- “ECCO_2_R” OR
- “extracorporeal CO_2_ removal” OR
- “interventional lung assist” OR
- “ila” OR
- “percutaneous Extracorporeal Lung Assist” OR
- “pecla” OR
- “novalung” OR
- “respiratory dialysis” OR
- “CO2 dialysis”.

**Electronic searches**

- Ovid platform: Medline, Scopus, (Embase)
- LILACS and ISI Web of Science from 1976 to current.
- Cochrane Central Database
- Clinical Trials.gov for on-going studies

**Searching other resources**

- Reference lists of included studies
- Reference lists of reviews

**Additional File C.** Search strategy.

The search strategy developed for this search is shown using the example of MEDLINE.

1 extracorporeal*.mp.

2 extra-corporeal*.mp.

3 "low flow".mp.

4 1 or 2 or 3

5 exp Carbon Dioxide/

6 "carbon dioxid*".mp.

7 "carbondioxid*".mp.

8 co2.mp.

9 "co 2".mp.

10 "gas exchange*".mp.

11 5 or 6 or 7 or 8 or 9 or 10

12 remov*.mp.

13 eliminat*.mp.

14 12 or 13

15 4 and 11 and 14

16 4 and decarboxyl*.mp.

17 ECCO2R*.mp.

18 EC-CO2R*.mp.

19 ECCO-2R*.mp.

20 ECCO2-R*.mp.

21 EC-CO-2R*.mp.

22 EC-CO2-R*.mp.

23 ECCO-2-R*.mp.

24 EC-CO-2-R*.mp.

25 17 or 18 or 19 or 20 or 21 or 22 or 23 or 24

26 (interventional adj5 "lung assist*").mp.

27 ((extracorporeal* or extra-corporeal*) adj5 "lung assist*").mp.

28 ((pumpless or pump-less) adj5 "lung assist*").mp.

29 (percutaneous adj5 "lung assist*").mp.

30 novalung.mp.

31 nova-lung.mp.

32 pecla.mp.

33 (ila and (assist or device* or extracorporeal or extra-corporeal or co2 or "co 2" or "carbon dioxid*" or "gas exchange" or decarboxyl*)).mp.

34 hemolung.mp.

35 hemo-lung.mp.

36 decapsmart.mp.

37 26 or 27 or 28 or 29 or 30 or 31 or 32 or 33 or 34 or 35 or 36

38 "respiratory dialysis".mp.

39 "co2 dialysis".mp.

40 "co 2 dialysis".mp.

41 'carbon dioxide dialysis'.mp.

42 38 or 39 or 40 or 41

43 15 or 16 or 25 or 37 or 42

44 limit 43 to yr="2000 -Current"

**Additional Table S1: Studies**

| **Study (Ref.)** | **Patients (n)**  **on ECCO_2_R** | **Device(s)** | **Type of study** | **Primary Clinical Goals** |
| --- | --- | --- | --- | --- |
| **ARDS** | **1179** |  |  |  |
| Liebold  2002 (45) | 70 | HE: AV PECLA | Cohort, retrospective | Improve gas exchange, more protective ventilation |
| Bein  2004 (37) | 30 | HE: AV PECLA | Cohort, retrospective | Improve gas exchange, more protective ventilation |
| Bein  2006 (4) | 90 | HE: AV ILA® | Cohort, retrospective | Improve gas exchange, more protective ventilation |
| Muellenbach  2008 (46) | 22 | HE: AV ILA® | Cohort, retrospective | Improve gas exchange, more protective ventilation |
| Floerchinger  2008 (39) | 159 | HE: AV ILA® | Cohort, prospective | Improve gas exchange, more protective ventilation |
| Zimmermann  2009 (21) | 51 | HE: AV ILA® | Cohort, prospective | Improve gas exchange, more protective ventilation |
| Weber-Carstens  2009 (22) | 10 | HE: AV ILA® | Cohort, retrospective | Improve gas exchange, more protective ventilation, weaning |
| Mueller  2009 (40) | 96 | HE: AV ILA® | Cohort, retrospective | Effects on gas exchange |
| Nierhaus  2011 (23) | 13 | HE: AV ILA® | Cohort, retrospective | Improve gas exchange, more protective ventilation |
| Forster  2013 (42) | 10 | LE: RRT + ECCO_2_R  (“LARRS“) | Cohort, prospective | Safety, effects on pH, ventilator settings, and hemodynamics. |
| Bein  2013 (20) | 40 (Control 39) | HE: AV ILA® | Randomized prospective trial | More protective ventilation |
| Ried  2013 (48) | 26 | HE: AV ILA® | Cohort, retrospective | Improve gas exchange |
| Allardet-Servant  2015 (25) | 11 | LE: RRT + ECCO_2_R (“Prismalung®”) | Cohort, prospective | Reduction of tidal volume |
| Munoz-Bendix  2015 (51) | 10 | HE: AV ILA® | Cohort, retrospective | Reduction of PaCO_2_ and ICP |
| Fanelli  2016 (26) | 15 | LE: Hemolung RAS® | Cohort, prospective | Reduction of tidal volume |
| Peperstraete  2017 (27) | 10 | LE: Abylcap® | Cohort, prospective | Reduction of PaCO_2_ |
| Winiszewski 1^a^  2018 (18) | 16 | LE: Hemolung RAS®, Prismalung®,  HE: AV ILA®, Cardiohelp HLS 5.0® | Cohort, retrospective | Reduction of tidal volume |
| Schmidt  2018 (29) | 20 | LE: Prismalung® | Cohort, prospective | Reduction of tidal volume |
| Combes  2019 (6) | 95 | LE: Hemolung RAS® (n=33)  HE: ILA Activve® (n=34)  HE: Cardiohelp HLS 5.0® (n=28) | Cohort, prospective | Reduction of tidal volume |
| Moerer  2019 (55) | 11 | LE: EQUA-smart® | Cohort, prospective | More protective ventilation |
| Augy 1^a^  2019 (19) | 24 | LE: Hemolung RAS® (n=18)  HE: ILA Activve® (n=6) | Cohort, prospective | More protective ventilation |
| Petran  2020 (57) | 73 | HE: AV ILA® | Cohort, retrospective | Improve gas exchange, more protective ventilation |
| Consales  2020 (30) | 22 | LE: Prismalung® | Cohort, retrospective | More protective ventilation,  facilitate weaning, avoid intubation (n=5) |
| Pestana  2020 (61) | 10 | LE: Prismalung® | Cohort, retrospective | More protective ventilation |
| McNamee  2021 (31) | 202 (Control 210) | LE: Hemolung RAS® | Randomized open-label prospective trial | Reduction of tidal volume |
| Ding  2021 (58) | 12 | LE: RRT + ECCO_2_R | Cohort, prospective | Improve gas exchange, more protective ventilation |
| Akkanti  2021 (63) | 31 | LE: Hemolung RAS® | Cohort retrospective | Improve gas exchange |
| **Bridge to LTX** | **44** |  |  |  |
| Fischer  2006 (38) | 12 | HE: AV ILA® | Cohort, prospective | Improve gas exchange, more protective ventilation |
| Ricci  2010 (62) | 12 | HE: AV ILA® (n=6)  LE: Decap Smart® (n=6) | Cohort, retrospective | Improve gas exchange |
| Schellongowski  2015 (8) | 20 | HE: AV ILA® (n=10)  HE: ILA Activve® (n=10) | Cohort, retrospective | Reduction of PaCO_2_ |
| **AECOPD** | **140** |  |  |  |
| Kluge  2012 (41) | 21 | HE: AV ILA® | Cohort, retrospective | Avoid intubation |
| Burki  2013 (49) | 7 | LE: Hemolung RAS® | Cohort, prospective | Avoid intubation |
| Del Sorbo  2015 (34) | 25 | LE: Decap Smart® | Cohort, prospective | Avoid intubation |
| Braune  2016 (35) | 25 | HE: ILA Activve® | Cohort, prospective | Avoid intubation |
| Winiszewski 2^a^  2018 (18) | 11 | LE: Hemolung RAS®, Prismalung®,  HE: AV ILA®, Cardiohelp HLS 5.0® | Cohort, retrospective | Reduction of PaCO_2_ |
| Augy 2^a^  2019 (19) | 30 | LE: Hemolung RAS® (n=25)  HE: ILA Activve® (n=5) | Cohort, prospective | Facilitate weaning (n = 21)  Avoid intubation (n = 9) |
| Diehl  2020 (56) | 12 | LE: Hemolung RAS® | Cohort, prospective | Improve gas exchange, reduce hyperinflation |
| Barrett  2022 (36) | 9 (Control 9) | LE: Hemolung RAS® | Randomized open-label prospective trial | Avoid intubation |
| **Mixed** | **298** |  |  |  |
| Arlt  2009 (47) | 20 | HE: AV ILA® | Cohort, retrospective | Improve gas exchange |
| Cho  2012 (24) | 11 | HE: AV ILA® | Cohort, retrospective | Improve gas exchange, more protective ventilation |
| Hermann  2014 (43) | 12 | HE: ILA Activve® | Cohort, retrospective | Improve gas exchange |
| Quintard  2014 (50) | 16 | LE: RRT + ECCO_2_R | Cohort, retrospective | Improve gas exchange |
| Tiruvoipati  2016 (52) | 15 | LE: Hemolung RAS® | Cohort, retrospective | Avoid intubation (n=5)  More protective ventilation (n=10) |
| Moss  2016 (59) | 14 | LE: Hemolung RAS® | Cohort, retrospective | Improve gas exchange |
| Hilty  2017 (28) | 20 | LE: ProLung® | Cohort, prospective | Avoid intubation (n=6)  More protective ventilation (n=14) |
| Seiler  2017 (53) | 24 | HE: Homburg Lung | Cohort, retrospective | Improve gas exchange |
| Cummins  2018 (54) | 60 | HE: AV ECCO_2_R (n=22)  HE: VV ECCO_2_R (n=38) | Cohort, retrospective | Improve gas exchange |
| Grasselli  2019 (44) | 11 | LE: ProLung® | Cohort, retrospective | Improve gas exchange |
| Nentwich  2019 (60) | 20 | LE: Prismalung® | Cohort, prospective | More protective ventilation |
| Inal  2021 (33) | 75 | LE: Decap Smart® | Cohort retrospective | Improve gas exchange, more protective ventilation |

HE: Higher extraction system, LE: Lower extraction system, ECCO_2_R: Extracorporeal carbon dioxide removal, ARDS: acute respiratory distress syndrome, COPD: chronic obstructive pulmonary disease, AECOPD: acute exacerbated chronic obstructive pulmonary disease, AKI: Acute kidney injury, LTX: lung transplantation, RRT: Renal replacement therapy, TBI: Traumatic brain injury, ICP: Intracranial pressure, BOS: Bronchiolitis obliterans syndrome, ARF: acute respiratory failure, MV: mechanical ventilation, CARDS: COVID-19 ARDS

^a^Separate analysis of the two subgroups because different clinical goals were followed for the ARDS and COPD group, respectively.

**Additional Table S2.** Devices designed for ECCO_2_R and their basic specifications.

| **Name** | **Manufacturer** | **Configuration** | **Catheter/Cannula** | **Pump** | **Gas exchange membrane surface** | **Blood Flow Range** | **Remarks** |
| --- | --- | --- | --- | --- | --- | --- | --- |
| **Higher extraction devices** | | | | | | | |
| ILA Membrane Ventilator^©^ | Novalung (now: Xenios), Germany | A-V  (referred to as pECLA,) | Novaport One  A: 13 - 15 F  V: 15 - 17 F | None. | 1.3 m^2^ | Ideally 800-1500 mL/min^a^ |  |
| ILA-Activve console | Novalung (Xenios), Germany | V-V | Novaport Twin  18 - 24 F  (or two single lumen cannulas) | Diagonal | ILA: 1.3 m^2^  Minilung: 0.65 m^2^  Minilung petite: 0.32 m^2^ | 18F: 800-1000 mL/min  22F: 800-2000 mL/min  24 F (femoral): 1000-2500 mL/min ^b^ | Platform: Xenios console. |
| PALP^©^ | Getinge, Germany | V-V | No specific cannula. | Centrifugal | 0.98 m^2^ | 200-2800 mL/min | Platform:  Cardiohelp console. |
| **Lower extraction devices** | | | | | | | |
| Prismalung^©^ | Baxter, USA | V-V | HD (13 - 14 F) | Roller | 0.32 m^2^ | Up to 450 mL/min |  |
| multiECCO_2_R | Fresenius Medical Care, Germany | V-V | HD (13 -14 F) | Roller | 1.35 m^2^ | 100-500 mL/min | Platform: Fresenius multiFiltrate |
| Prolung^©^ | Estor, Italy | V-V | HD (13 -14 F) | Roller | 1.8 m^2^ | Up to 450 mL/min |  |
| Abylcap^©^ | Bellco, Italy | V-V | HD (13 - 14 F) | Roller | 0.67 m^2^ | Up to 450 mL/min |  |
| Decap Smart^©^ (Plus) | Medica, Italy | V-V | HD (13 - 14 F) | Roller | 0.33 m^2^-1.35 m^2^ (depends on membrane) | Up to 450 mL/min |  |
| Hemolung^©^ | A-Lung, USA | V-V | Hemolung DL catheter (15.5 F) | Centrifugal | 0.59 m^2^ | 350-550 mL/min |  |

ILA: Interventional lung assist; PECLA: Pumpless extracorporeal lung assist; PALP: Pump Assisted Lung Protection; A-V: Arterio-venous; V-V: Veno-venous; HD: Haemodialysis catheter; F: French; ECMO: Extracorporeal membrane oxygenation

^a^ Blood flow dependent on cannula size and arterio-venous pressure gradient, ^b^ Estimated range dependent on preload and cannula position

**Additional Table S3.** Available data from included studies.

| **First Author** | **Year of  Publication** | **P_plat_/PIP** | **PEEP** | **V_T_** | **RR** | **PaCO_2_** | **P/F Ratio** | **pH** | **Complications  reported** |
| --- | --- | --- | --- | --- | --- | --- | --- | --- | --- |
| Liebold | 2002 | 0 | 0 | 0 | 0 | + | + | 0 | + |
| Bein | 2004 | + | + | + | + | + | + | 0 | + |
| Bein | 2006 | + | + | + | + | + | + | + | + |
| Fischer | 2006 | + | + | 0 | 0 | + | + | + | + |
| Muellenbach | 2008 | + | + | + | 0 | + | + | + | + |
| Flörchinger | 2008 | 0 | + | + | + | + | + | + | + |
| Zimmermann | 2009 | + | + | + | + | + | + | + | + |
| Weber-Carstens | 2009 | + | + | + | + | + | + | + | + |
| Arlt | 2009 | 0 | 0 | 0 | 0 | + | + | 0 | + |
| Mueller | 2009 | 0 | 0 | 0 | 0 | + | + | + | 0 |
| Ricci | 2010 | 0 | 0 | 0 | 0 | + | 0 | + | 0 |
| Nierhaus | 2011 | + | + | + | + | + | + | + | + |
| Kluge | 2012 | 0 | 0 | 0 | + | + | + | + | + |
| Cho | 2012 | + | + | + | + | + | + | + | + |
| Forster | 2013 | + | + | + | + | + | 0 | + | + |
| Burki | 2013 | 0 | 0 | 0 | 0 | + | 0 | + | + |
| Ried | 2013 | 0 | 0 | 0 | 0 | + | + | + | + |
| Bein | 2013 | 0 | 0 | + | 0 | 0 | + | 0 | + |
| Hermann | 2014 | 0 | 0 | + | + | + | + | + | + |
| Quintard | 2014 | + | 0 | + | 0 | + | + | + | + |
| Allardet-Servant | 2015 | + | + | + | + | + | + | + | + |
| Del Sorbo | 2015 | 0 | 0 | 0 | + | + | + | + | + |
| Munoz-Bendix | 2015 | + | + | 0 | + | + | + | + | + |
| Schellongowski | 2015 | 0 | + | + | 0 | + | + | + | + |
| Fanelli | 2016 | + | + | + | + | + | + | + | + |
| Braune | 2016 | 0 | 0 | 0 | + | + | 0 | + | + |
| Tiruvoipati | 2016 | + | 0 | 0 | 0 | + | 0 | + | + |
| Moss | 2016 | 0 | 0 | 0 | 0 | + | 0 | + | + |
| Peperstraete | 2017 | + | + | + | + | + | + | + | + |
| Hilty | 2017 | + | + | + | + | + | + | + | + |
| Seiler | 2017 | 0 | 0 | 0 | 0 | + | + | + | + |
| Winiszewski a | 2018 | + | + | + | + | + | + | + | + |
| Winiszewski b | 2018 | 0 | + | + | + | + | + | + | + |
| Schmidt | 2018 | + | + | + | + | + | + | + | + |
| Cummins | 2018 | 0 | + | 0 | + | + | + | + | + |
| Combes | 2019 | + | + | + | + | + | + | + | + |
| Grasselli | 2019 | 0 | + | 0 | + | + | + | + | + |
| Moerer | 2019 | 0 | + | + | + | + | 0 | 0 | + |
| Nentwich | 2019 | + | 0 | + | 0 | + | 0 | + | + |
| Augy a | 2019 | 0 | 0 | + | 0 | + | 0 | + | + |
| Augy b | 2019 | 0 | 0 | + | 0 | + | 0 | + | + |
| Petran | 2020 | + | + | + | 0 | + | + | + | + |
| Consales | 2020 | + | + | + | + | + | + | + | + |
| Pestana | 2020 | 0 | 0 | 0 | 0 | + | + | + | + |
| Diehl | 2020 | + | + | 0 | 0 | + | 0 | + | + |
| McNamee | 2021 | + | + | + | + | + | + | + | + |
| Wohlfarth | 2021 | + | + | + | + | + | + | + | + |
| Inal | 2021 | + | + | + | + | + | + | + | + |
| Ding | 2021 | + | 0 | 0 | 0 | + | 0 | 0 | 0 |
| Akkanti | 2021 | 0 | 0 | + | + | + | 0 | + | + |
| Barrett | 2022 | 0 | 0 | 0 | + | + | 0 | + | + |

+

P_plat_: Plateau pressure, cmH_2_O PIP: Peak inspiratory pressure, cmH_2_O, PEEP: Positive end-expiratory pressure, cmH_2_O, V_T_: Tidal volume, mL/kg RR: Respiratory rate, breaths/min, PaCO_2_, mmHg: Arterial partial pressure of carbon dioxide

**Additional Table S4.** Adverse Events from observational studies.

|  |  |  | Clinically significant | | |
| --- | --- | --- | --- | --- | --- |
| Trial | Sample size | All adverse events | Bleeding events | Thrombotic or ischaemic events | Technical adverse events |
| **Higher extractors** |  |  |  |  |  |
| Forster 2013 | 10 | 0 | 0 | 0 | 0 |
| Burki 2013 | 7 | 4 | 2 | 1 | 1 |
| Quintard 2014 | 16 | 0 | 0 | 0 | 0 |
| Allardet-Servant 2015 | 11 | 2 | 0 | 1 | 1 |
| Del Sorbo 2015 | 25 | 13 | 4 | 7 | 2 |
| Fanelli 2016 | 15 | 2 | 0 | 0 | 1 |
| Tiruvoipati 2016 | 15 | 14 | 7 | 1 | 0 |
| Moss 2016 | 14 | 4 | 1 | 1 | 1 |
| Peperstraete 2017 | 10 | 10 | 5 | 3 | 2 |
| Hilty 2017 | 20 | 0 | 0 | 0 | 0 |
| Schmidt 2018 | 20 | 2 | 2 | 0 | 0 |
| Grasselli 2019 | 11 | 13 | 1 | 11 | 0 |
| Moerer 2019 | 11 | 0 | 0 | 0 | 0 |
| Nentwich 2019 | 20 | 0 | 0 | 0 | 0 |
| Consales 2020 | 22 | 0 | 0 | 0 | 0 |
| Pestana 2020 | 10 | 0 | 0 | 0 | 0 |
| Diehl 2020 | 12 | 6 | 3 | 3 | 0 |
| Inal 2021 | 75 | 0 | 0 | 0 | 0 |
| Akkanti 2021 | 31 | 2 | 1 | 1 | 0 |
| McNamee 2021 | 202 | 50 | 36 | 5 | 9 |
| Barrett 2022 | 9 | 4 | 3 | 0 | 1 |
| *Subgroup pooled rate (95%CI)* |  | *0.21  (0.14 to 0.32)* | *0.04  (0.02 to 0.08)* | *0.09  (0.05 to 0.16)* | *0.02  (0.01 to 0.04)* |
|  |  |  |  |  |  |
| **Lower extractors** |  |  |  |  |  |
| Liebold 2002 | 70 | 18 | 0 | 16 | 1 |
| Bein 2004 | 30 | 13 | 3 | 6 | 4 |
| Bein 2006 | 90 | 23 | 4 | 13 | 0 |
| Fischer 2006 | 12 | 0 | 0 | 0 | 0 |
| Muellenbach 2008 | 22 | 5 | 1 | 2 | 1 |
| Flörchinger 2008 | 159 | 20 | 0 | 13 | 2 |
| Zimmermann 2009 | 51 | 6 | 1 | 4 | 0 |
| Weber-Carstens 2009 | 10 | 0 | 0 | 0 | 0 |
| Arlt 2009 | 20 | 10 | 0 | 8 | 1 |
| Nierhaus 2011 | 13 | 3 | 1 | 0 | 2 |
| Kluge 2012 | 21 | 11 | 10 | 0 | 0 |
| Cho 2012 | 11 | 11 | 2 | 8 | 1 |
| Ried 2013 | 26 | 6 | 5 | 1 | 0 |
| Hermann 2014 | 12 | 3 | 1 | 2 | 0 |
| Munoz-Bendix 2015 | 10 | 0 | 0 | 0 | 0 |
| Schellongowski 2015 | 20 | 9 | 2 | 6 | 0 |
| Braune 2016 | 25 | 25 | 8 | 12 | 3 |
| Seiler 2017 | 24 | 3 | 0 | 2 | 1 |
| Cummins 2018 | 60 | 18 | 10 | 3 | 3 |
| Petran 2020 | 73 | 2 | 2 | 0 | 0 |
| Bein 2013 | 40 | 3 | 0 | 3 | 0 |
| *Subgroup pooled rate (95%CI)* |  | *0.10  (0.04 to 0.25)* | *0.05  (0.02 to 0.12)* | *0.03  (0.01 to 0.08)* | *0.03 (0.01 to 0.06)* |
|  |  |  |  |  |  |
|  |  |  |  |  |  |
| **Mixed methods** |  |  |  |  |  |
| Winiszewski 2018 | 26 | 26 | 1 | 5 | 0 |
| Combes 2018 | 95 | 56 | 13 | 13 | 5 |
| Augy 2019 | 54 | 54 | 19 | 11 | 8 |
| Wohlfarth 2021 | 11 | 5 | 1 | 2 | 1 |
| *Subgroup pooled rate (95%CI)* |  | *0.77  (0.57 to 1.00)* | *0.14  (0.06 to 0.33)* | *0.17  (0.12 to 0.24)* | *0.07  (0.03 to 0.15)* |
|  |  |  |  |  |  |
| *Overall pooled rate (95%CI)* |  | *0.19  (0.12 to 0.28)* | *0.05  (0.03 to 0.08)* | *0.07  (0.04 to 0.11)* | *0.02  (0.01 to 0.04)* |

**Additional Figure S1**: Risk of bias assessment (Robins-I tool)


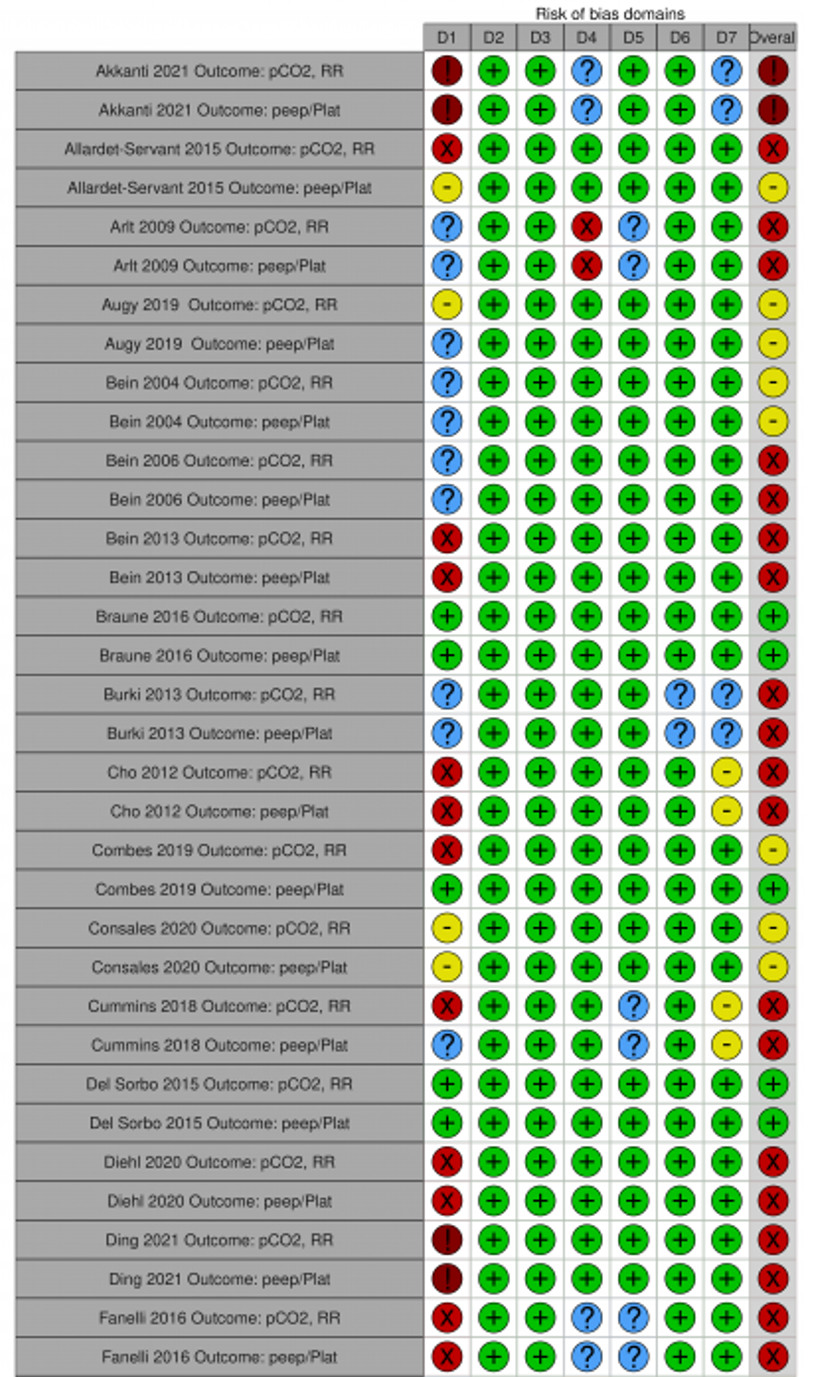


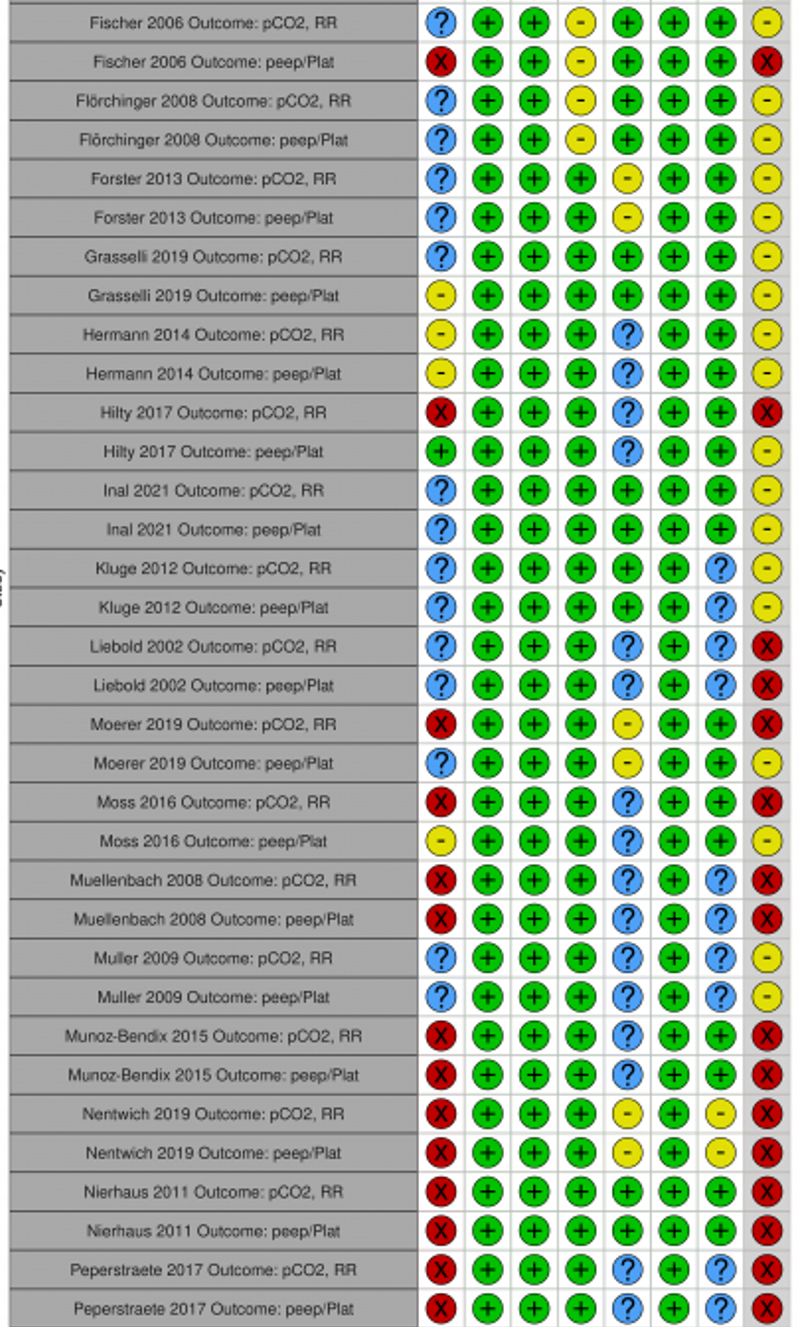


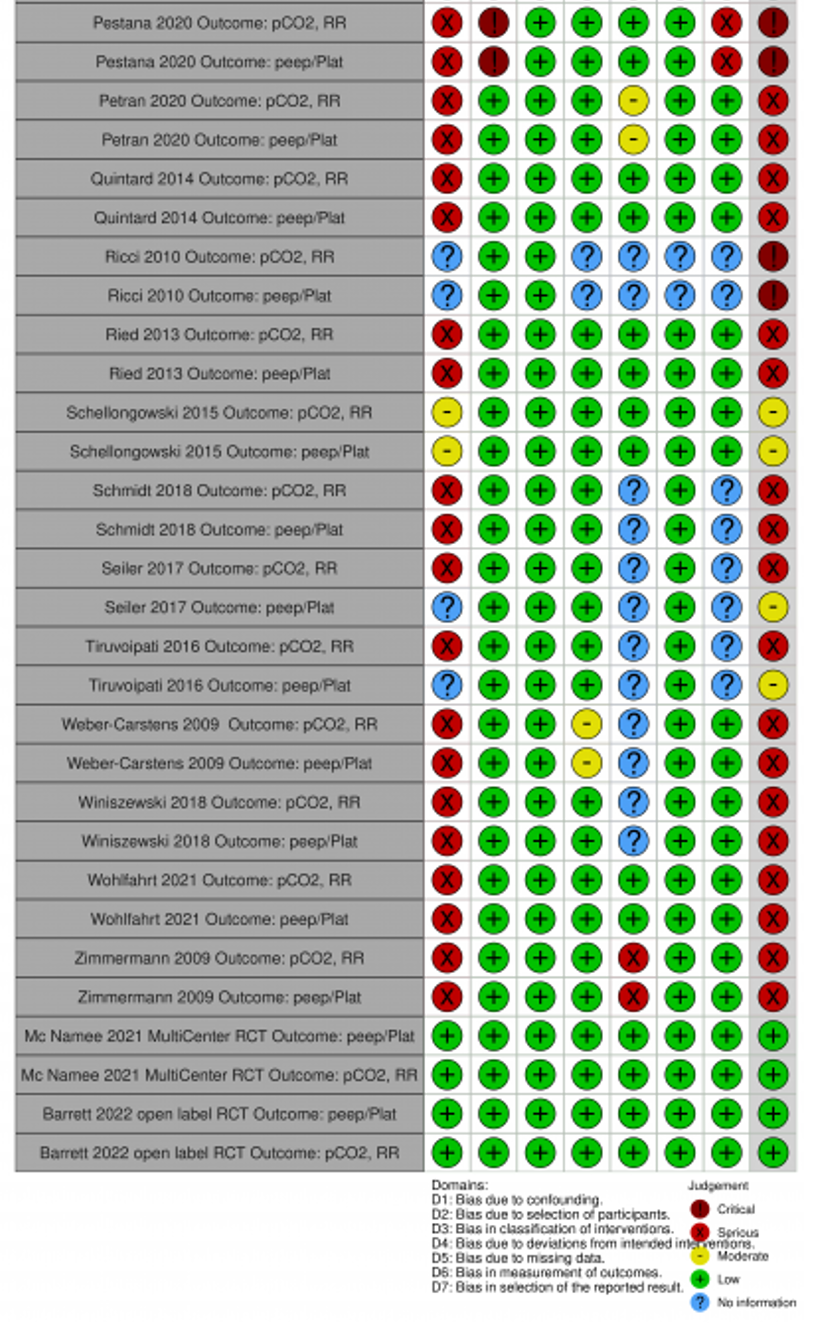


**Additional Figure S2 a, and b**: Change of (a) PaCO_2_, mmHg and (b) pH within 24 hours after initiating ECCO_2_R (all studies)


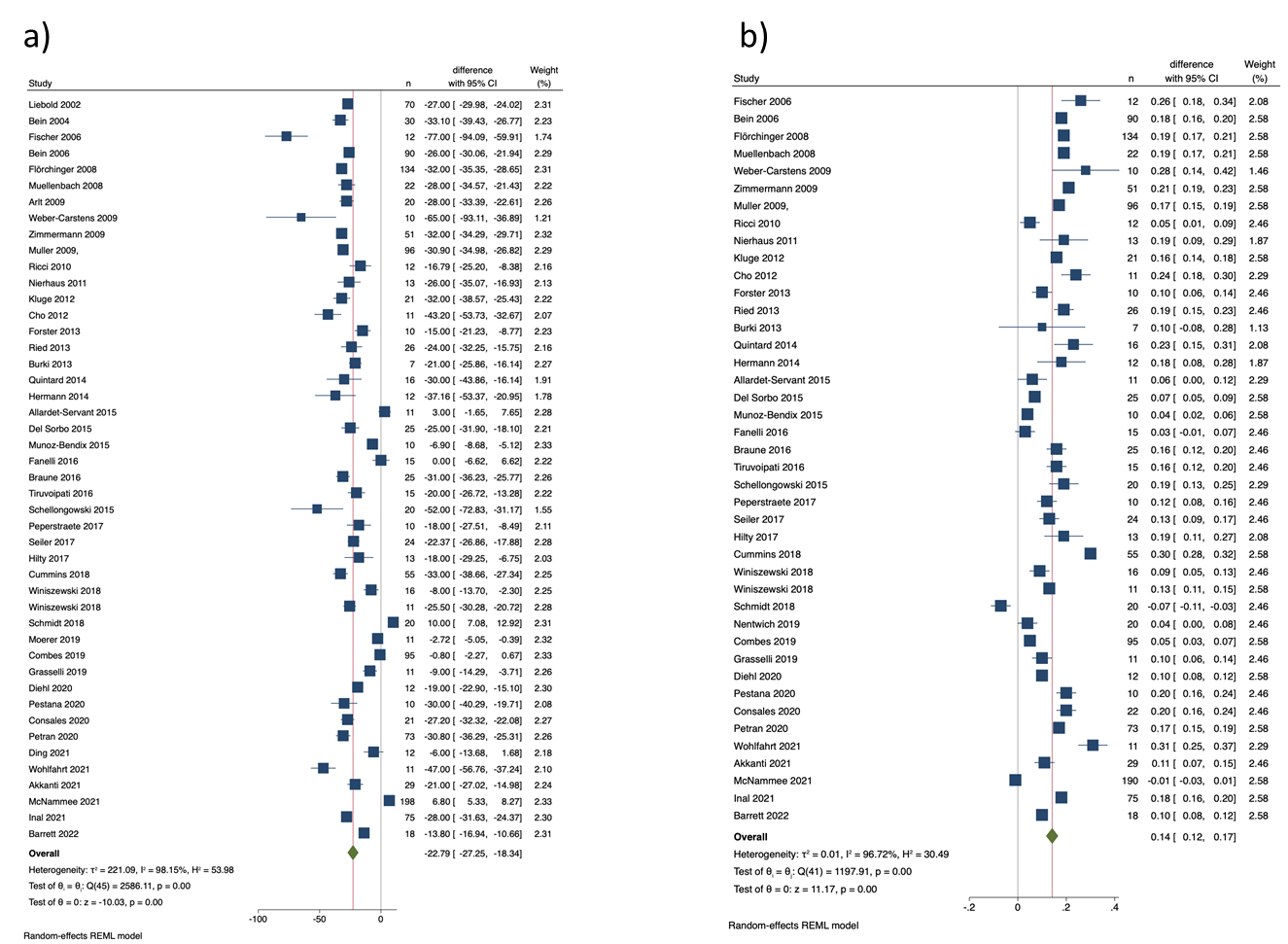


**Additional Figure S3 a, and b.** Change of (a) PaO_2_/FiO_2_ ratio, mmHg and (b) PEEP, cmH_2_O within 24 hours after initiating ECCO_2_R (all studies)


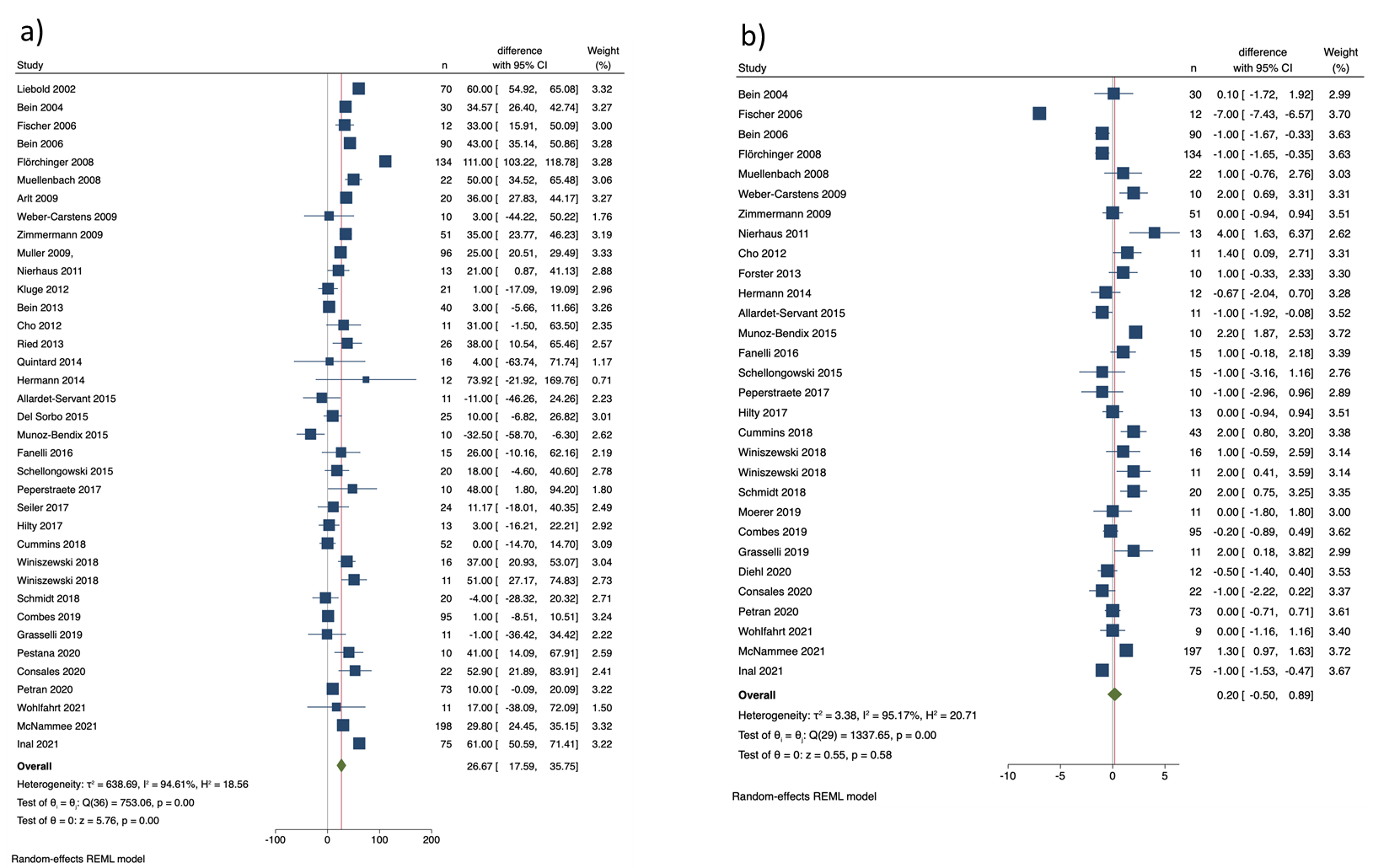


**Additional Figure S4 a, b, and c:** Change of (a) plateau pressure, cmH_2_O, (b) tidal volume, mL, and (c) respiratory rate, breaths/min within 24 hours after initiating ECCO_2_R (all studies)


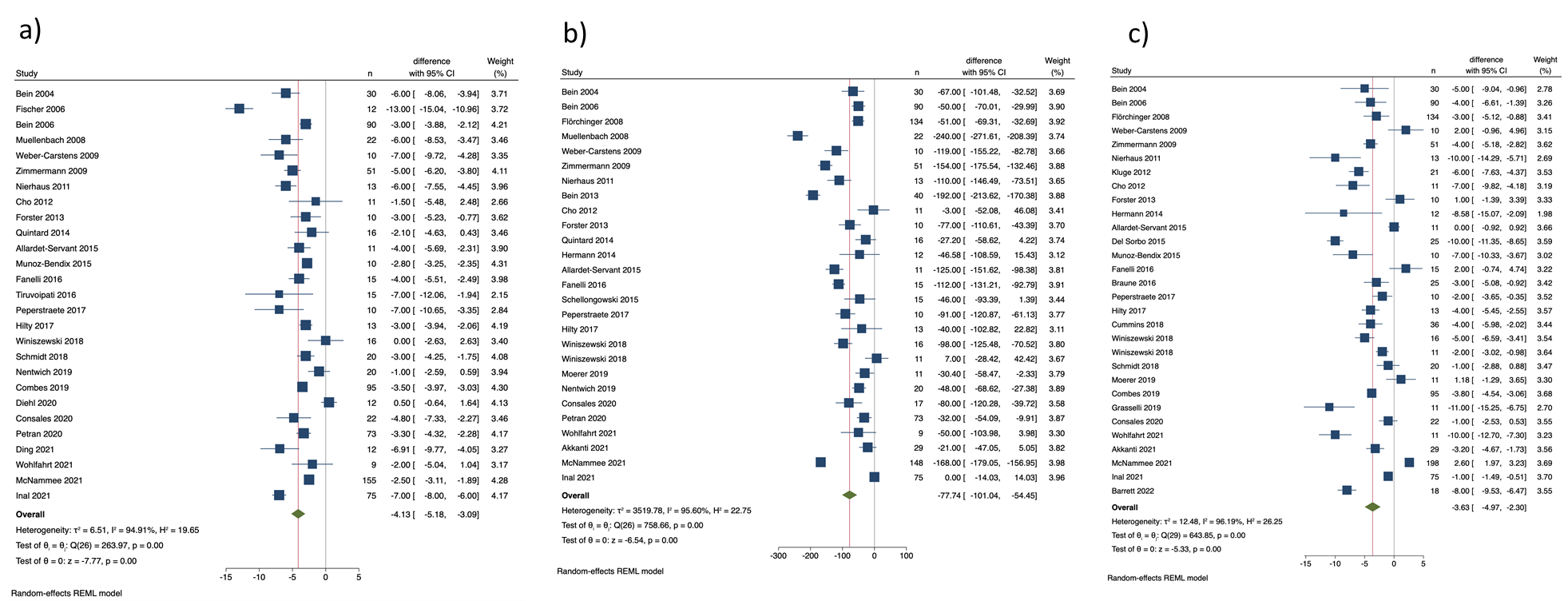


**Additional Figure S5 a, and b.** Change of (a) PaO_2_/FiO_2_ ratio, mmHg and (b) PEEP, cmH_2_O within 24 hours after initiating ECCO_2_R (diagnoses subgroups)


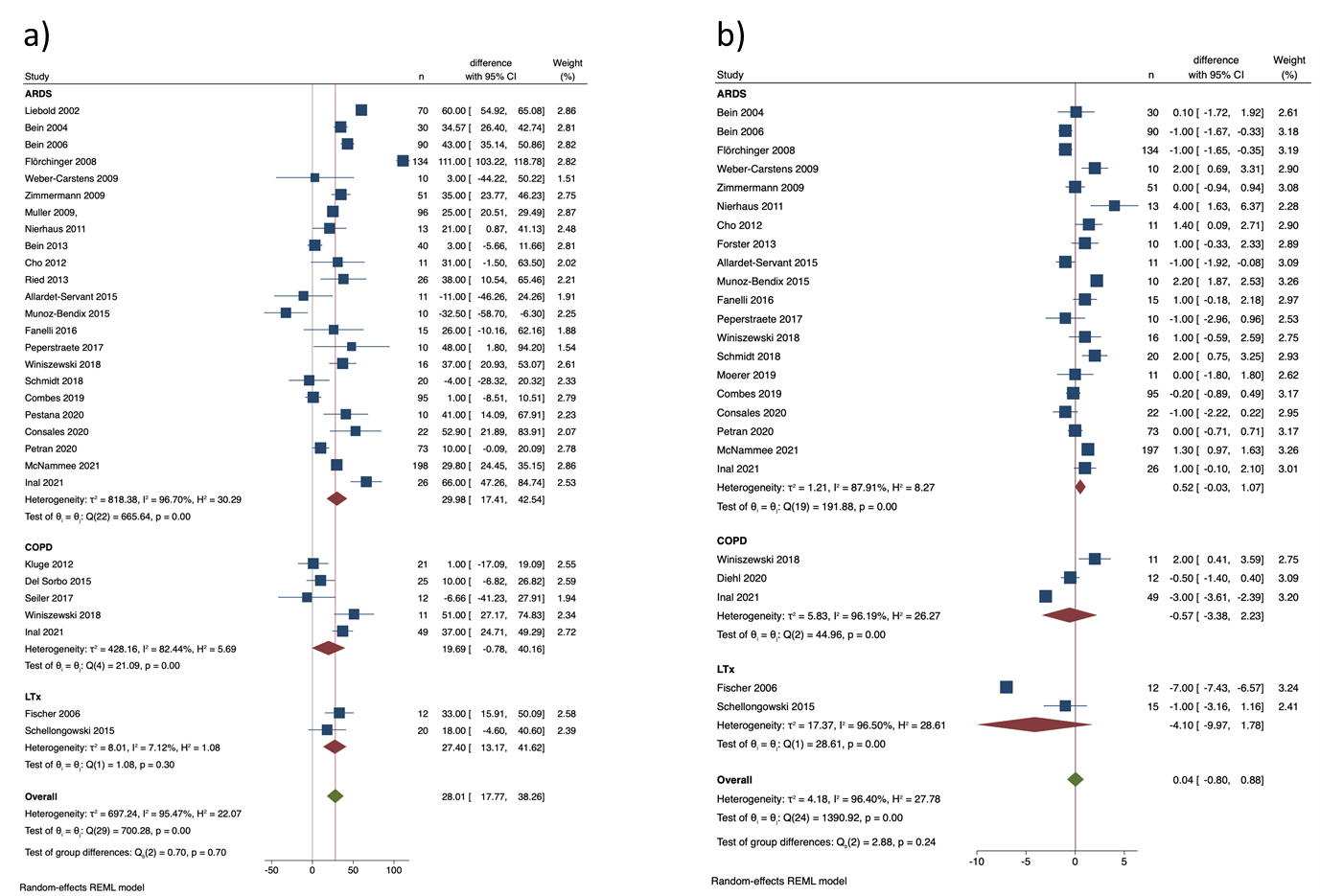


**Additional Figure S6 a, and b.** Change of (a) PaCO_2_, mmHg and (b) PaO_2_/FiO_2_ ratio, mmHg within 24 hours after initiating ECCO_2_R according to diagnosis and extraction.


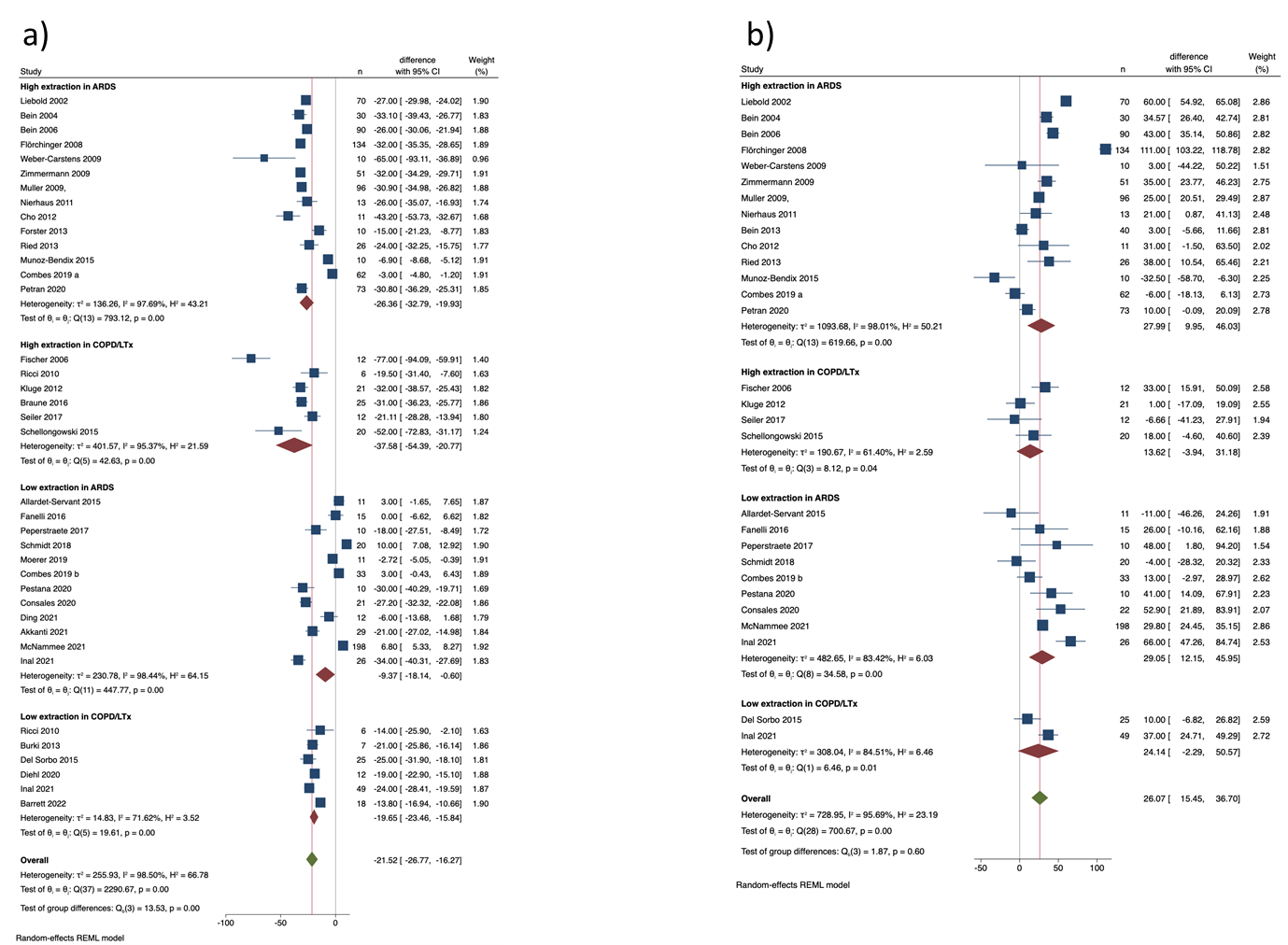


**Additional Figure S7 a, and b.** Change of (a) PaO_2_/FiO_2_ ratio, mmHg and (b) PEEP, cmH_2_O within 24 hours after initiating ECCO_2_R (lower extraction and higher extraction subgroups).


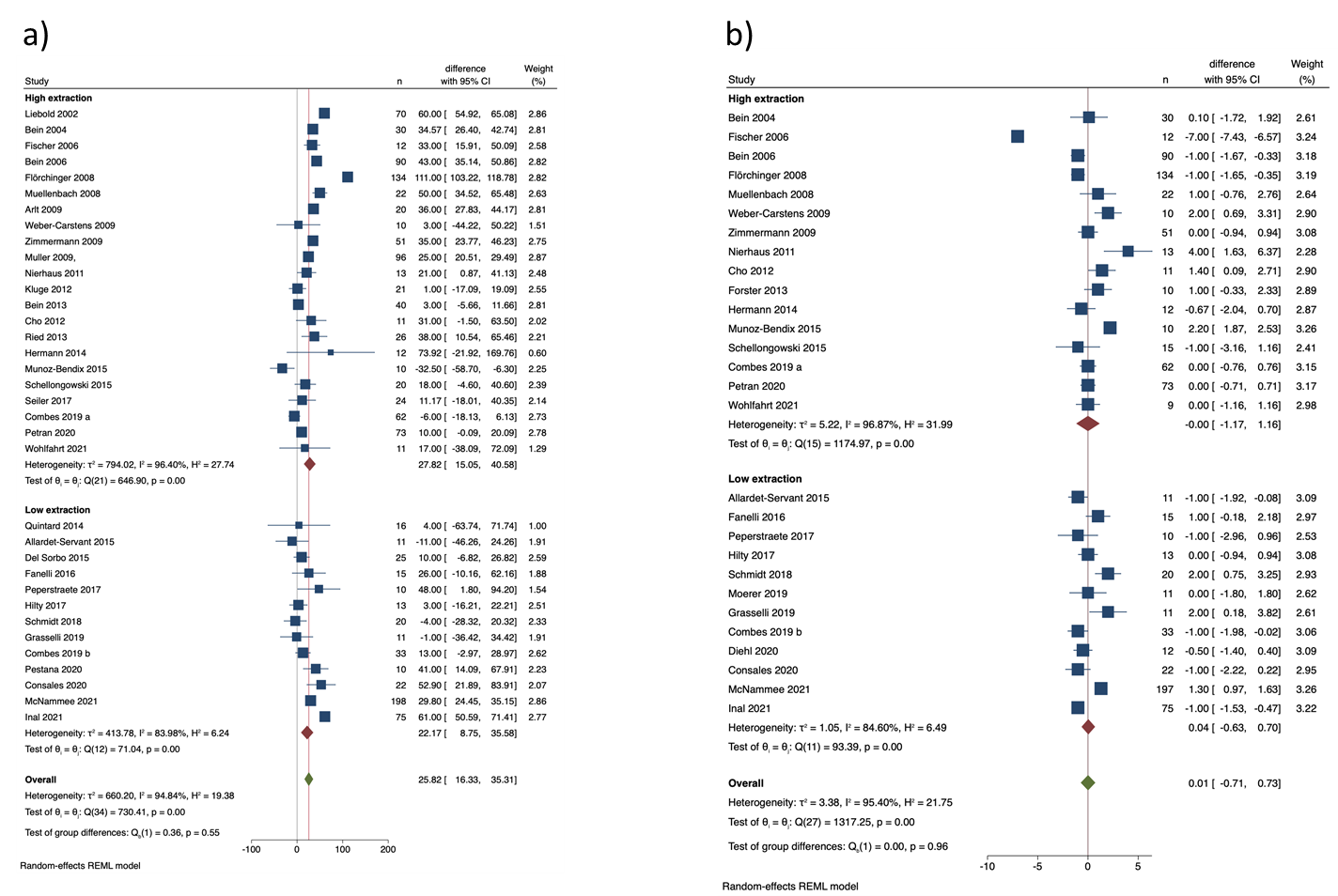


**Additional Figure S8 a, and b.** Change of (a) PaO_2_/FiO_2_ ratio, mmHg and (b) PEEP, cmH_2_O within 24 hours after initiating ECCO_2_R according to diagnosis and extraction


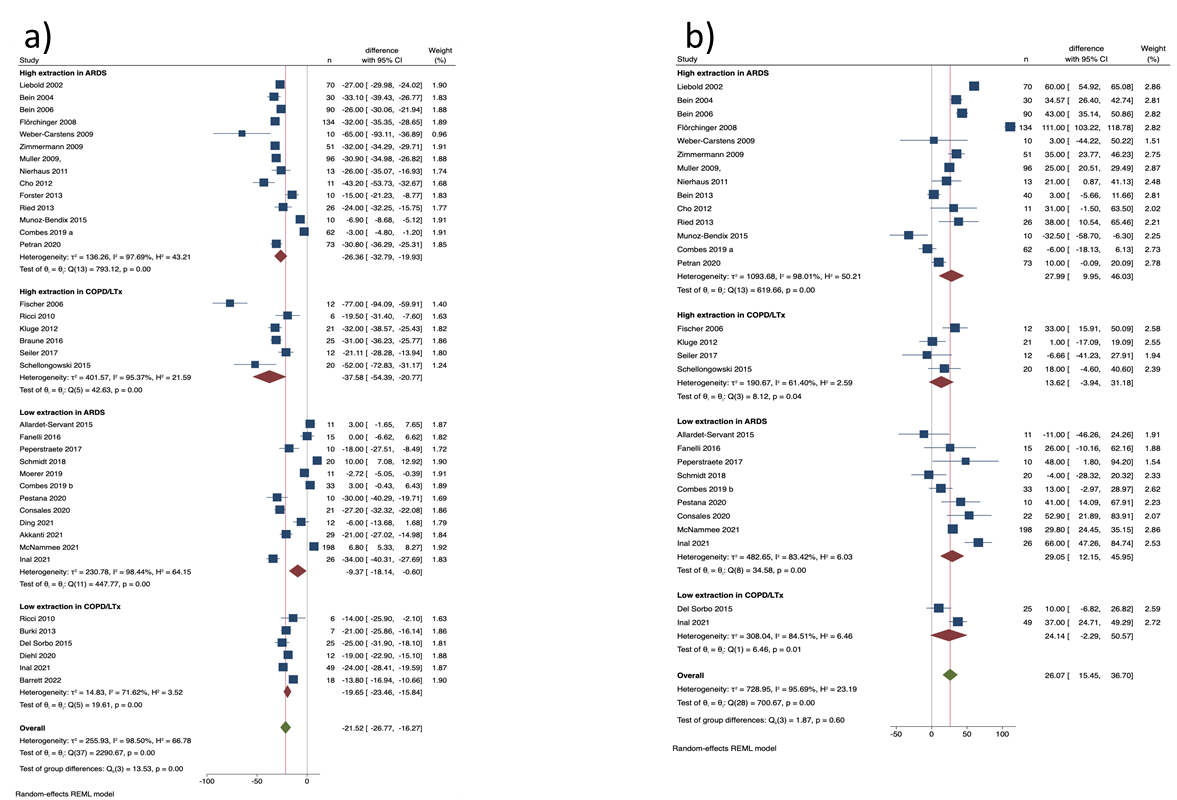


**Additional Figure S9 a, and b:** Change of (a) plateau pressure cmH_2_O and (b) tidal volume mL within 24 hours after initiating ECCO_2_R (lower extraction and higher extraction subgroups).

**
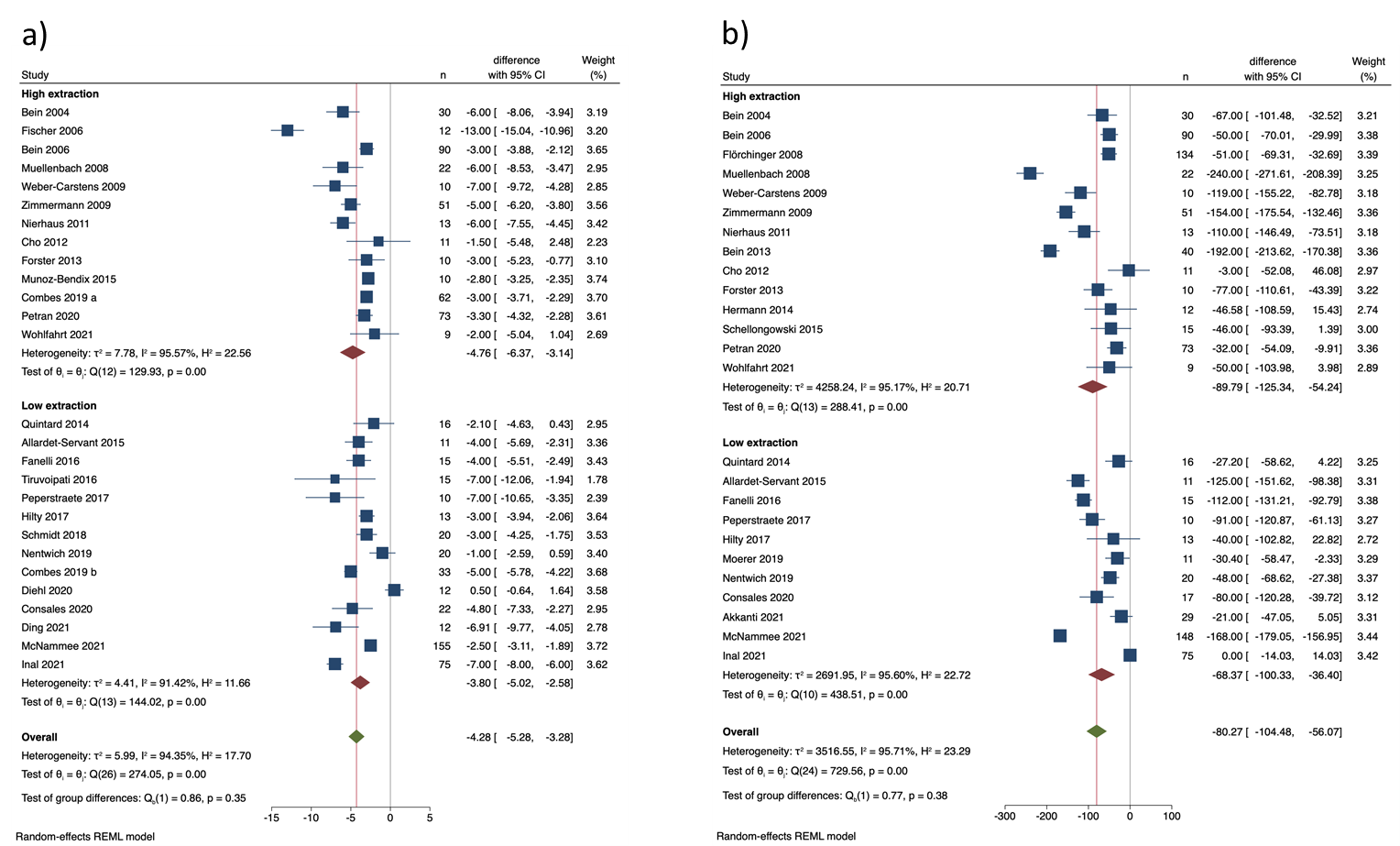
**

**Additional Figure S10 a, b, and c:** Change of (a) plateau pressure, cmH_2_O, (b) tidal volume, mL, and (c) respiratory rate, breaths/min within 24 hours after initiating ECCO_2_R according to diagnosis and extraction.


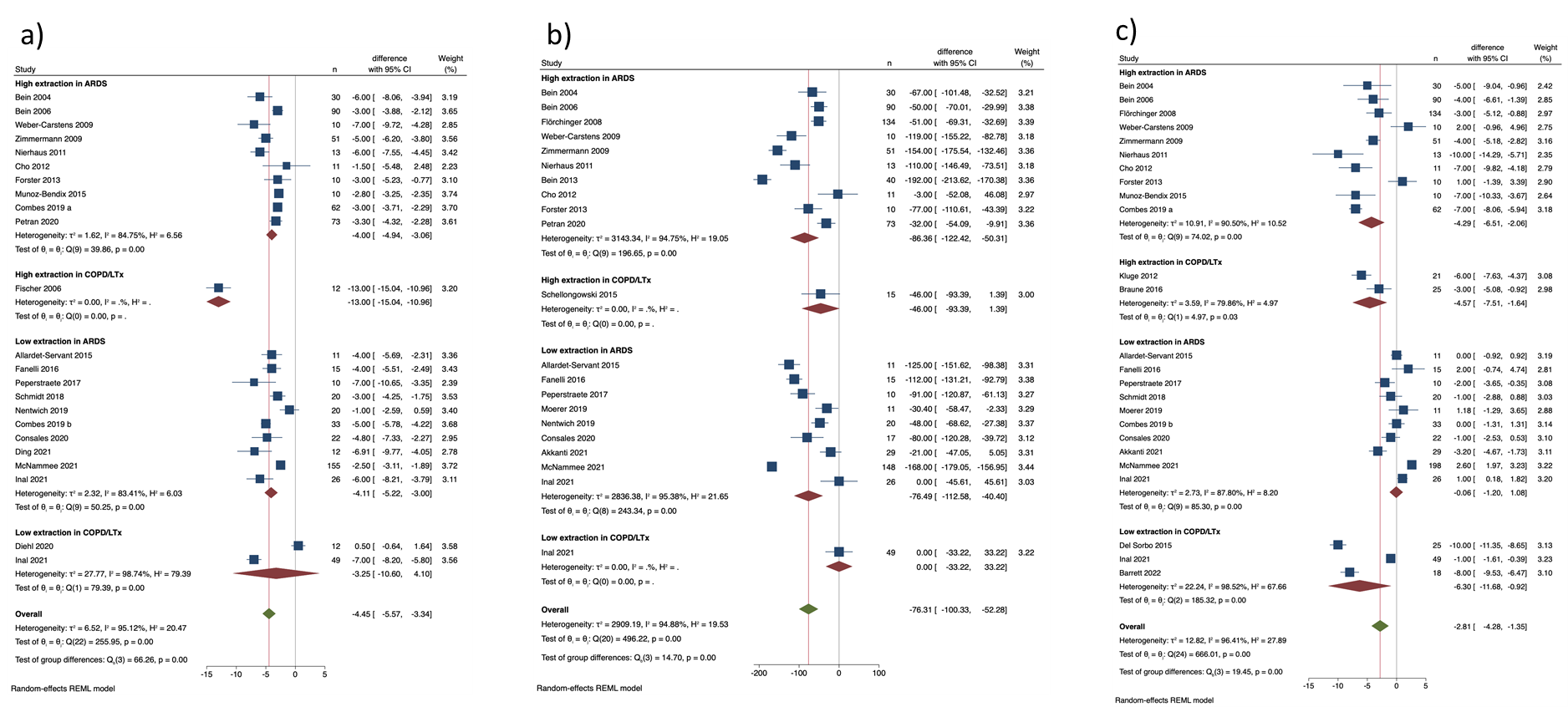


**Additional Figure S11 a-g.** (a) PaCO_2_, mmHg, (b) pH, (c) PaO_2_/FiO_2_ ratio, mmHg, (d) PEEP, cmH_2_O, (e) plateau pressure, cmH_2_O, (f) tidal volume, mL, and (g) respiratory rate, breaths/min according to risk of bias.


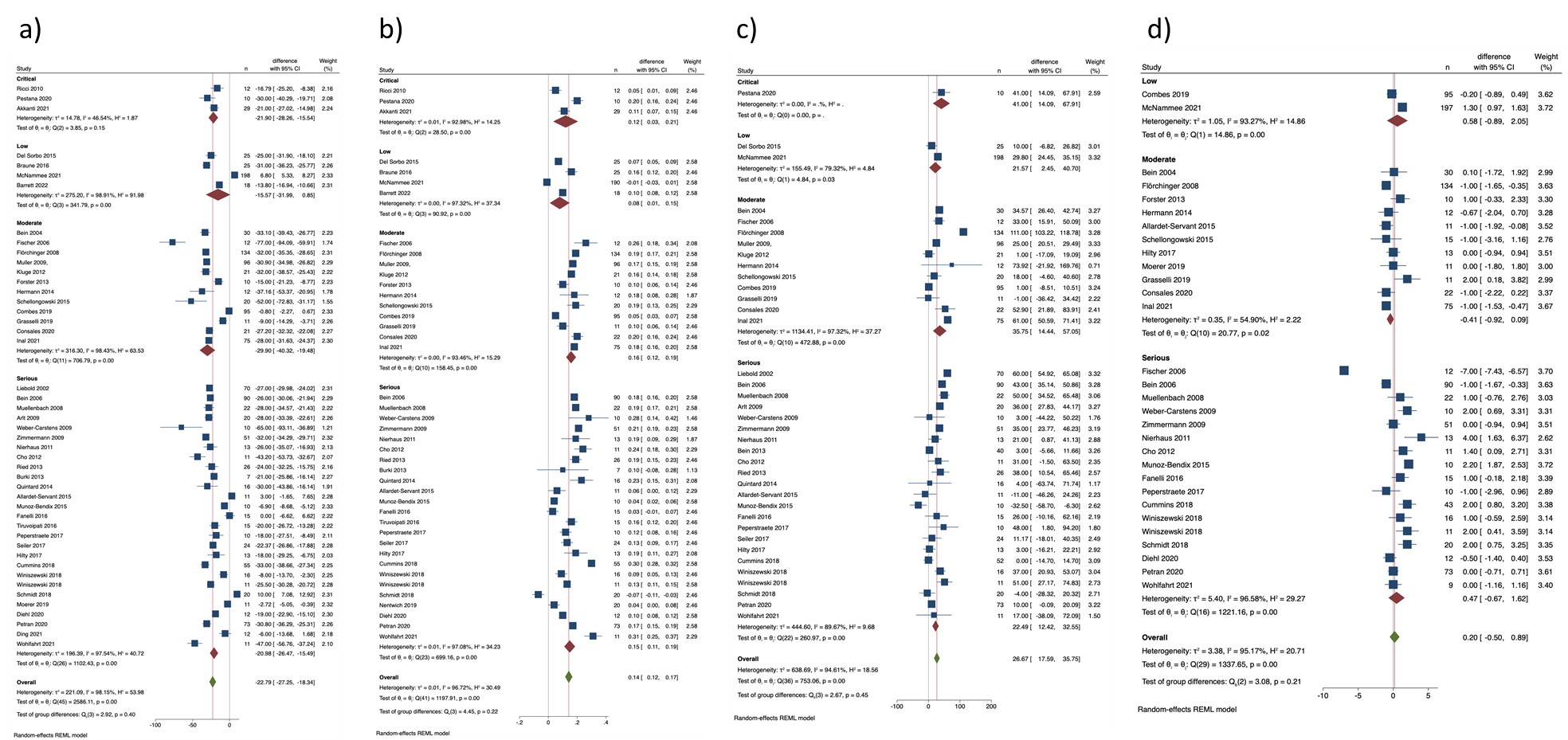


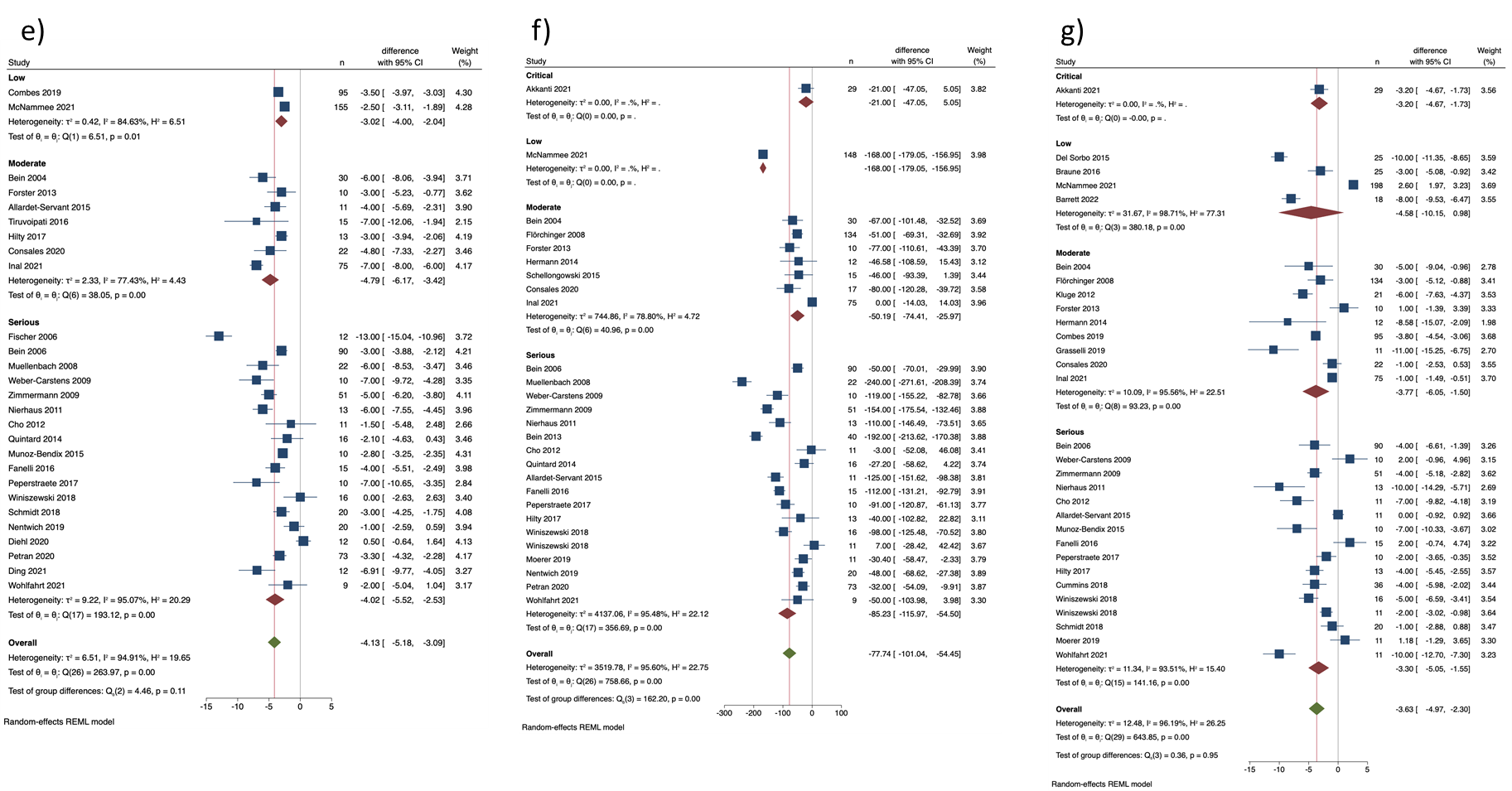

Supplement: Supplementary file 1 — Additional file 1. Additional File A. PRISMA 2020 checklist. Additional File B. Criteria for considering studies for this review. Additional File C. Search strategy. Additional Table S1. Studies. Additional Table S2. Devices designed for ECCO2R and their basic specifications. Additional Table S3. Available data from included studies. Additional Table S4. Adverse Events from observational studies. Additional Figure S1: Risk of bias assessment (Robins-I tool). Additional Figure S2 a, and b: Change of (a) PaCO2, mmHg and (b) pH within 24 hours after initiating ECCO2R (all studies). Additional Figure S3 a, and b: Change of (a) PaO2/FiO2 ratio, mmHg and (b) PEEP, cmH2O within 24 hours after initiating ECCO2R (all studies). Additional Figure S4 a, b, and c: Change of (a) plateau pressure, cmH2O, (b) tidal volume, mL, and (c) respiratory rate, breaths/min within 24 hours after initiating ECCO2R (all studies). Additional Figure S5 a, and b: Change of (a) PaO2/FiO2 ratio, mmHg and (b) PEEP, cmH2Owithin 24 hours after initiating ECCO2R (diagnoses subgroups). Additional Figure S6 a, and b: Change of (a) PaCO2, mmHg (a) and (b) PaO2/FiO2 ratio, mmHg within 24 hours after initiating ECCO2R according to diagnosis and extraction. Additional Figure S7 a, and b: Change of (a) PaO2/FiO2 ratio, mmHg and (b) PEEP, cmH2O within 24 hours after initiating ECCO2R (lower extraction and higher extraction subgroups). Additional Figure S8 a, and b: Change of (a) PaO2/FiO2 ratio, mmHg and (b) PEEP, cmH2O within 24 hours after initiating ECCO2R according to diagnosis and extraction. Additional Figure S9 a, and b: Change of (a) plateau pressure, cmH2O and (b) tidal volume, mL within 24 hours after initiating ECCO2R (lower extraction and higher extraction subgroups). Additional Figure S10 a, b, and c: Change of (a) plateau pressure, cmH2O, (b) tidal volume, mL, and (c) respiratory rate, breaths/min within 24 hours after initiating ECCO2R according to diagnosis and extraction. Additional Figure S11 [file 13054_2024_4927_MOESM1_ESM.docx]
